# White-light emission from discrete heterometallic lanthanide-directed self-assembled complexes in solution†
†Electronic supplementary information (ESI) available. See DOI: 10.1039/c7sc00739f



**DOI:** 10.1039/c7sc00739f

**Published:** 2017-03-06

**Authors:** Oxana Kotova, Steve Comby, Christophe Lincheneau, Thorfinnur Gunnlaugsson

**Affiliations:** a School of Chemistry , Trinity Biomedical Sciences Institute (TBSI) , Trinity College Dublin , Dublin 2 , Ireland . Email: combys7@gmail.com ; Email: gunnlaut@tcd.ie

## Abstract

White-light-emitting materials have attracted significant interest in recent years due to their potential applications in solid-state lighting and flat-panel displays. Design of such materials is challenging and often relies on the use of multiple fluorophores despite the fact that single component systems yield materials with enhanced stability and reproducibility. Herein, we have developed a white-light-emitting system based on the formation of discrete lanthanide-based self-assembled complexes using a newly-designed ligand. We demonstrate that fine tuning of the lanthanide ions molar ratio in the self-assemblies combined with the intrinsic blue fluorescence of the ligand allows for the successful emission of pure white light with CIE coordinates of (0.33, 0.34).

## Abstract 2

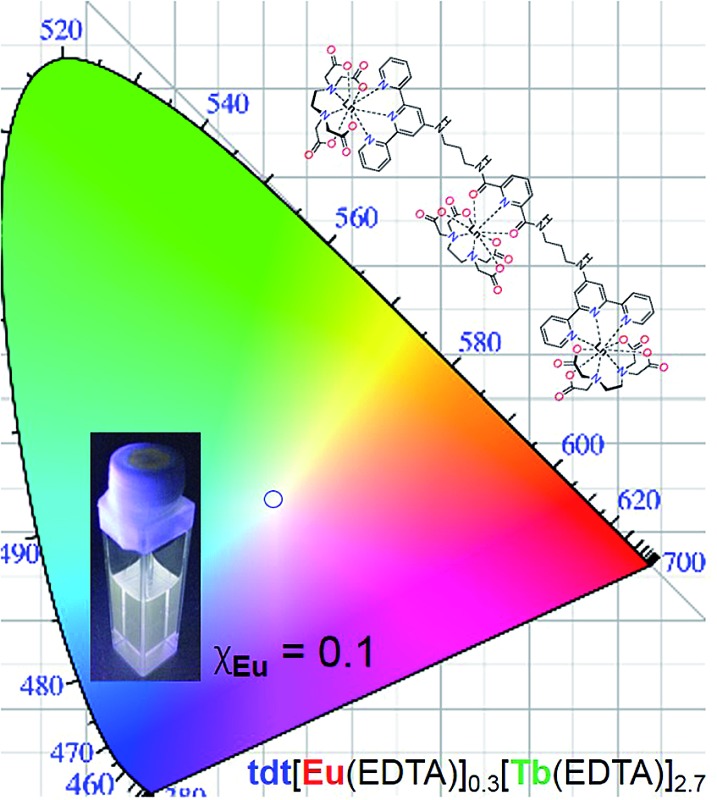
Herein, we have developed a white-light-emitting system based on the formation of discrete lanthanide-based self-assembled complexes using a newly-designed ligand. We demonstrate that fine tuning of the lanthanide ions molar ratio in the self-assemblies combined with the intrinsic blue fluorescence of the ligand allows for the successful emission of pure white light with CIE coordinates of (0.33, 0.34).

## Introduction

**Fig. 1:**
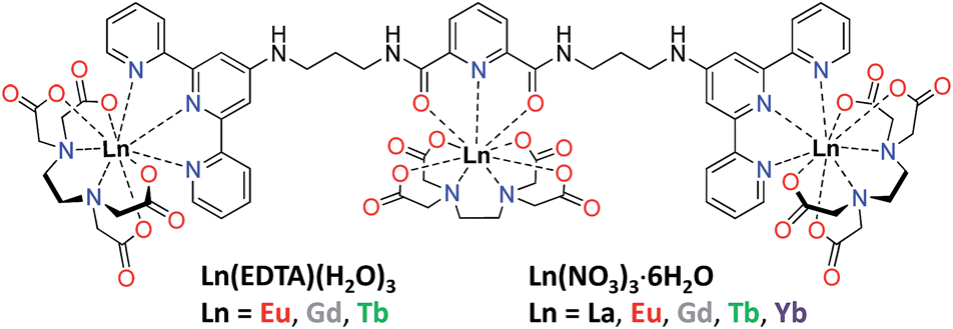
Structural formula of multidentate **tdt** ligand composed of dpa and two tpy units free for binding Ln(EDTA)·(H_2_O)_3_ or Ln(NO_3_)_3_·6H_2_O.

The interest in the design of white-light emissive materials mainly stems from their potential application as emissive layers in white organic light-emitting diodes (WOLEDs) for general solid-state lighting and flat-panel display backlights.^1,2^ Amongst inorganic and organic materials there are different approaches being developed by researchers in order to achieve efficient white light emission.^2a,3^ In the case of organic molecules traditional strategies for the design of white light emitters are based on (1) the mixing of several fluorophores emitting either blue, red and green or orange and turquoise light which when combined cover the entire visible spectrum or (2) the use of single molecule emitting white light.^4^ The latter approach has been much less developed to date although it provides materials with enhanced stability and reproducibility, as well as avoiding the reabsorption of the blue light by the red and green components. Moreover, the use of a single molecule or single component systems simplifies the fabrication of thin films for planar emissive devices such as WOLEDs.^1*f*^ To achieve white-light-emitting single molecules, one can use lanthanide (Ln) complexes by combining blue emission from an organic molecule, or ligand, and the unique luminescent properties of Ln ions,^5,6^ in particular the red and green line-like emission from Eu(iii) and Tb(iii), respectively. Among Ln complexes that have been used for generating white-light-emitting materials, the first examples used d- and/or f-metals such as a bimetallic iridium and Eu(iii) system^7^ or heteropentanuclear Al(iii)–Eu(iii) complexes.^8^ The use of ternary complexes with Eu(tta)_3_ (tta = 1,1,1-trifluoro-3-(2-thenoyl)acetone) and coumarin–rhodamine^9^ or pyrazolyl-triazine ligands have also been developed.^10^ Recently, Ward and co-workers used a Eu(iii)–DO3A (DO3A = 1,4,7-tris(carboxymethyl)-1,4,7,10-tetraazacyclododecane) complex, possessing a naphthalimide antenna, to obtain white-light emission *via* controllable solvent-dependent aggregation.^11^ Mixed-Ln(iii) complexes including metal–organic frameworks (MOFs) have also been utilised in the production of white-light emitters^12^ and this approach has now been taken further with the successful formation of white-light-emitting supramolecular gels.^13a–e^ Another approach consists in the synthesis of homo- and hetero-Ln-grafted polymers, achieved by copolymerisation of individual Ln complexes.^13f–k^ Despite the above developments, to the best of our knowledge, the design of white-light emitters based solely on the directed self-assembly of f-metal ions in solution has not been achieved to date. Herein we developed novel discrete white-light-emitting Ln-based heterometallic assemblies in solution, using the multidentate ligand **tdt** shown in Fig. 1. The ligand structure comprises a central dipicolinate (dpa) unit connected to two 2,2′:6′,2′′-terpyridyl moieties (tpy), providing three tridentate binding sites (one NO_2_ + two N_3_) suited for the coordination of Ln ions.^14,15^ We have previously used the tpy moiety for sensitisation of Eu(iii) emission in f–d metal ion based conjugates^16^ as well as for the formation of extended 3D network in luminescent gels.^17^ Both the dpa and tpy moieties have been shown to be efficient energy harvesters for the sensitisation of the Eu(iii)- and Tb(iii)-centred emission, making these highly attractive for incorporation into a single ligand design, such as **tdt**, for white-light-emitting materials.^13d,17a,18^

## Results and discussion

### Synthesis, characterisation and optical properties of ligand **tdt**

**Scheme 1:**
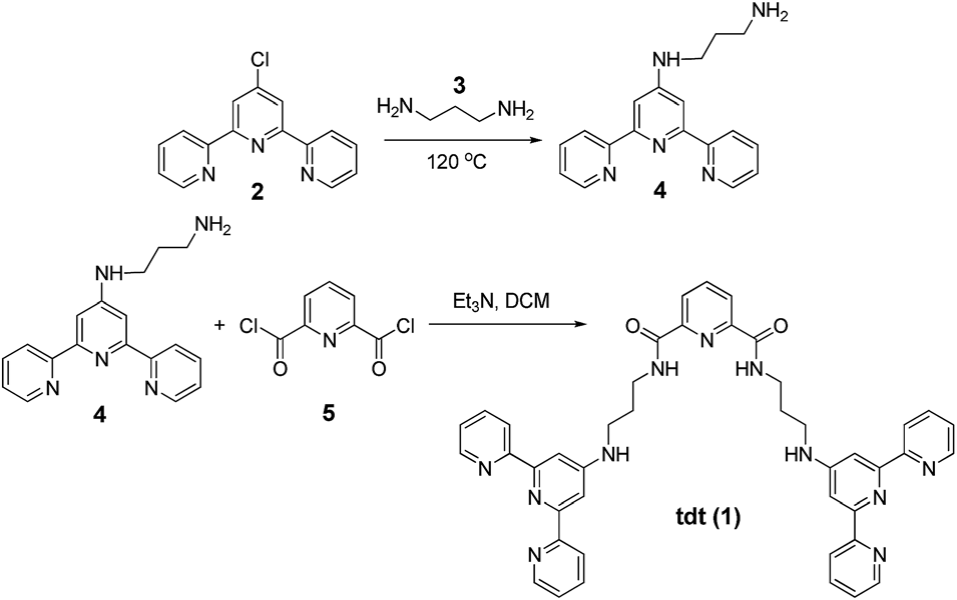
General synthetic procedure of **tdt** ligand.

**Fig. 2:**
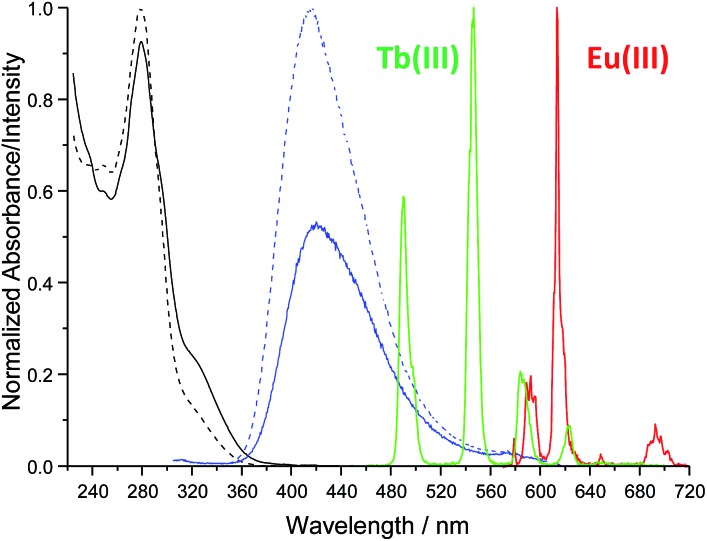
Absorption and emission (*λ*_ex_ = 280 nm) spectra of **tdt** (5 μM) in the absence ( <svg xmlns="http://www.w3.org/2000/svg" version="1.0" width="16.000000pt" height="16.000000pt" viewBox="0 0 16.000000 16.000000" preserveAspectRatio="xMidYMid meet"><metadata> Created by potrace 1.16, written by Peter Selinger 2001-2019 </metadata><g transform="translate(1.000000,15.000000) scale(0.005147,-0.005147)" fill="currentColor" stroke="none"><path d="M0 1440 l0 -80 360 0 360 0 0 80 0 80 -360 0 -360 0 0 -80z M1040 1440 l0 -80 360 0 360 0 0 80 0 80 -360 0 -360 0 0 -80z M2080 1440 l0 -80 320 0 320 0 0 80 0 80 -320 0 -320 0 0 -80z"/></g></svg> ) and presence (—) of Ln(iii) ions in methanol at 298 K.

The synthesis of **tdt** was achieved in two steps (Scheme 1); the *N*-[2,2′:6′,2′′]terpyridin-4′-yl-propane-1,3-diamine (**4**) was first obtained following a previously reported synthetic procedure and subsequently reacted with the dpa central unit using a standard peptide coupling procedure described by Fuller *et al.* ^19^ to obtain the target ligand as a white powder in approximately 67% yield. The ^1^H NMR spectrum (600 MHz, (CD_3_)_2_SO; Fig. S1–S6, ESI†) of ligand **tdt** showed the presence of the central pyridine protons as a set of two resonances at *δ* = 8.00–7.70 ppm, indicative of a *C*_2_ symmetry for **tdt**. The presence of amide bonds was confirmed by the broad signals occurring at *δ* = 8.78 and 7.02 ppm as well as by the N–H vibrations observed in the IR spectrum at *ca.* 3200 cm^–1^; the elemental analysis also confirmed the formation of the desired structure. The UV-visible absorption spectrum of **tdt** (5 μM) in methanol solution displayed a broad band centred at approximately 280 nm (log *ε*_280_ = 4.83) and two shoulders at 294 and 320 nm (Fig. 2). These were all assigned to tpy π → π* transitions and, for the 280 nm band to some extent to similar transitions occurring within the dpa unit. Excitation of these transitions gave rise to a single broad fluorescence emission band in the blue region with *λ*_max_ at 415 nm (Fig. 2 and S7, ESI†). It is noteworthy that the ligand-centred blue emission displayed its maximum intensity at 4–5 μM, with any increase of the concentration above 10 μM resulting in a decrease of both the fluorescence emission and lifetime due to aggregation *via* intermolecular π–π stacking interactions (Fig. S8 and S9, ESI†). The occurrence of these interactions was confirmed further by the change in shape and red-shifted maxima which were observed in the excitation spectra upon increase of the **tdt** concentration (Fig. S10, ESI†).^20^

### Interaction of **tdt** with Ln(NO_3_)_3_·6H_2_O (Ln(iii) = La, Eu, Gd, Tb, Yb)

The interaction of **tdt** with Eu(iii) and Tb(iii) ions was studied in methanol solution by monitoring the changes in the absorption and fluorescence spectra of **tdt** as well as those observed in the Eu(iii)- and Tb(iii)-centred emission (Fig. S11–S13, ESI† for Eu(iii)). Upon addition of Eu(iii), the absorption of **tdt** displayed a marked hypochromic effect at 278 nm (–10%) with a concomitant increase in the absorbance at 292 nm (+6%) and 322 nm (+86%), as shown in Fig. 2. These changes occurred mainly within the first equivalent added and were attributed to the binding of Eu(iii) to one or several of the tridentate binding sites of **tdt**; this clearly confirming the coordinating ability of this ligand toward Ln(iii) ions. The observation of two pseudo-isosbestic points at *ca.* 237 and 288 nm further indicated the presence of at least two metallic species in equilibrium in solution. The existence of multiple species in solution was further corroborated by the changes observed in the ligand-centred fluorescence emission upon excitation at 280 nm (Fig. S12, ESI†). The broad emission band centred at *ca.* 415 nm was significantly affected upon binding to Eu(iii) ions. Initially, from 0 → 0.5 equivalents of Eu(iii), the emission was strongly quenched (–44%), while displaying a 5 nm red-shift (Fig. 2). This quenching was a direct consequence of complex formation and concomitant sensitisation of metal-centred emission. Briefly, the **tdt** ligand acts as an antenna, populating the ^5^D_0_ excited state of Eu(iii) *via* energy transfer, after which deactivation to the ^7^F_*J*_ ground states (*J* = 0–4) yields the characteristic Eu(iii) line-like emission at 580, 595, 616, 650, and 696 nm (Fig. 2 and S13, ESI†). However, after the initial quenching, the ligand fluorescence increased slightly between 0.5 → 1.0 equivalents before displaying a significant enhancement, with the final emission intensity becoming almost twice that of the initial **tdt** fluorescence. In contrast, the Eu(iii)-centred emission displayed the reverse trend to that seen for the ligand singlet excited state, reaching a maximum intensity before 0.5 equivalents after which the emission decreased rapidly up to the addition of 1 equivalent and more slowly thereafter. These changes in both the ligand and the Eu(iii)-centred emissions are in agreement with the successive formation of f-metal ion complexes with various stoichiometries during the course of the titration. The same overall trend was observed in the absorption, fluorescence and metal-centred emission spectra when Tb(NO_3_)_3_ was used. As shown in Fig. 2, **tdt** can also act as an efficient sensitizer for Tb(iii), thereby giving rise to the characteristic emission bands that are centred at 491, 545, 584, 623, 647, 668 and 679 nm, attributed to the ^5^D_4_ → ^7^F_*J*_ (*J* = 6–0) transitions, respectively. The results of these titrations suggested the successive formation of three main metal-based species in solution: the first species characterised by a weaker ligand fluorescence and a strong Ln-centred emission; a second one giving rise to similar fluorescence intensities, while the lanthanide emission was much weaker; and finally, the third species displaying an enhanced ligand fluorescence, but a weak Ln(iii) emission. Despite these observations, no satisfying fit of the spectroscopic data could be obtained when the overall changes in the absorption, fluorescence and Ln(iii)-centred emission were analysed using the nonlinear regression analysis program SPECFIT.^21^ The evolving factor analysis revealed, however, the presence of four absorbing or fluorescent species in solution, and three for the Ln(iii)-centred emission. Unfortunately, the exact Ln : **tdt** stoichiometry of these species could not be determined with certainty. A closer look at the changes in the absorption spectra of **tdt** upon addition of Ln(iii) revealed that the hyperchromic effect observed at 322 nm upon binding to the Ln(iii) ion was more pronounced for Tb(iii) than for Eu(iii). Hence, further UV-visible titrations were performed using La(iii) and Yb(iii); the results demonstrated that the size of the Ln(iii) ion played a significant role in dictating the distribution of the species in solution, potentially giving rise to the formation of species with different Ln : L stoichiometric ratios (Fig. S14, ESI†). While ditopic ligands containing tpy have been known to assemble into monometallic and dynamic polymetallic assemblies in solution,^22a–c^ the fact that the linker, in the case of **tdt**, was also able to coordinate Ln(iii) ions brought another level of complexity to the overall self-assembly processes.^22*d*^ As a result, we moved towards the use of Ln(EDTA)·(H_2_O)_3_ complexes instead of the Ln(iii) salts to afford a better control over the self-assembly in solution.

### Interaction of **tdt** with Ln(EDTA)·(H_2_O)_3_ (Ln(iii) = Eu, Tb)

**Fig. 3:**
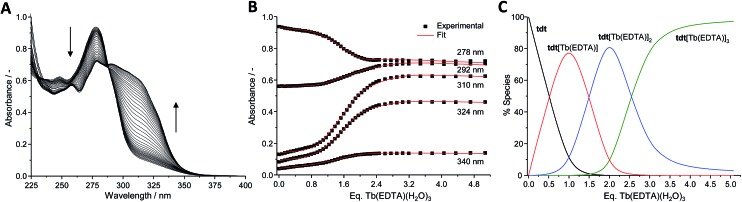
(A) Absorption spectra, (B) experimental binding isotherms and corresponding fits (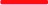), (C) speciation–distribution diagram for the UV-visible titration of **tdt** (12 μM) with Tb(EDTA)·(H_2_O)_3_ in methanol at 298 K.

The **tdt**, with its three tridentate binding sites, is perfectly suited to host up to three hexadentate Ln(iii) complexes such as Ln(EDTA)·(H_2_O)_3_. To confirm this, UV-visible absorption titrations of **tdt** with Ln(EDTA)·(H_2_O)_3_ (Ln(iii) = Eu, Tb) were performed in methanol. The evolution of the absorption spectrum of **tdt** upon addition of both Eu(iii) and Tb(iii) EDTA complexes (Fig. 3 and S15, ESI†) pointed towards the formation of three different metallic species during the course of the titration. Fitting the spectroscopic data using non-linear regression analysis, provided the exact stoichiometries of these species in solution, *i.e.* Ln (EDTA)  : **tdt** 1 : 1, 2 : 1 and 3 : 1, as well as their binding constants (Table S1, ESI†).

The fluorescence emission arising from the successive formation of the homometallic assemblies with Ln(EDTA)·(H_2_O)_3_ showed that the Eu(iii) emission reached 87% of its maximum intensity within the addition of the first equivalent. Simultaneously, the blue ligand fluorescence increased regularly throughout the titration. However, it never became the major contributor to the overall emission (*ca.* 39% maximum at 3 equivalents, Fig. S16, ESI†). Conversely, the corresponding homometallic assemblies formed with Tb(EDTA)·(H_2_O)_3_ showed quite a different behaviour. The Tb(iii) emission reached only 56% of its maximum emission at 1 equivalent, while the ligand fluorescence appeared stronger and became a major contributor to the overall emission after the addition of 3 equivalents of Tb(EDTA)·(H_2_O)_3_ (Fig. S17, ESI†). The excitation spectra for these systems confirmed that both chromophoric units (dpa, tpy) present in **tdt** were involved in the sensitisation of the Ln(iii) emission. Interestingly, the lowest energy band (which belongs to the tpy units) seemed not to be affected by the addition of the first equivalent of Ln(EDTA)·(H_2_O)_3_, but showed a marked 18 nm red-shift thereafter (Fig. S18, ESI†). This can be explained by the different nature of the binding sites available for coordination within the **tdt** ligand design. Due to the hard Lewis acid nature of the Ln(iii) ions, the central NO_2_ binding site of the dpa unit should provide a stronger coordination than the N_3_ binding sites of the terminal tpy units, and consequently, dpa is likely to be the favoured coordination site for the first equivalent of Ln(EDTA)·(H_2_O)_3_. Further evidence for the binding to **tdt** and saturation of the first coordination sphere of the Ln(iii) ions in these assemblies was achieved by comparing their emission profiles (absolute and relative intensities) with those of Ln(EDTA)·(H_2_O)_3_ and Ln(NO_3_)_3_·6H_2_O, respectively. The overall profile of the emission spectra for the ternary assemblies, **tdt**[Ln(EDTA)]_3_, and particularly the relative intensities of the Eu(iii) ^5^D_0_ → ^7^F_*J*_ and Tb(iii) ^5^D_4_ → ^7^F_*J*_ transitions are clearly different to those observed for the corresponding EDTA complexes or Ln(iii) salt solutions (Fig. S19A, S20A and Tables S2, S3, ESI†). Moreover, the much stronger emission intensity observed when Ln(EDTA)·(H_2_O)_3_ was added to a solution of **tdt** compared to the same solutions in the absence of **tdt** provided another clear indication of the formation of luminescent **tdt**[Ln(EDTA)]_3_ ternary assemblies in solution (Fig. S19B and 20B, ESI†). Having demonstrated the formation of luminescent homometallic **tdt**[Ln(EDTA)]_3_ assemblies in solution, we next evaluated the possibility to obtain white light emission by mixing ligand-centred blue fluorescence with the characteristic red and green emission of Eu(iii) and Tb(iii).

### Heterometallic assemblies for white-light emission

Heterometallic assemblies, with the formula **tdt**[Eu(EDTA)]_*x*_[Tb(EDTA)]_3–*x*_, were prepared in solution through the successive addition of (i) Eu(EDTA)·(H_2_O)_3_ and (ii) Tb(EDTA)·(H_2_O)_3_. The sequence of additions was determined based on the behaviour observed for the homometallic assemblies, where dpa has been shown to be the most favoured binding site in **tdt**, while being at the same time a better sensitizer for Eu-centred emission than the tpy units. As a first attempt, 1 equivalent of Eu(EDTA)·(H_2_O)_3_ and 2 equivalents of Tb(EDTA)·(H_2_O)_3_ were added to a solution of **tdt** (12 μM) in methanol to form **tdt**[Eu(EDTA)][Tb(EDTA)]_2_, which appeared slightly pink-purple to the naked eye under excitation at 280 nm. The emission and excitation spectra of each individual intermediate species in the formation of the final ternary heterometallic assembly have been recorded and analysed to obtain CIE coordinates which agreed with the colour observed by naked eye, with CIE coordinates of (0.32, 0.24) determined for the final assembly (Fig. S21, ESI†). The good match between the UV-visible absorption and the excitation spectra clearly demonstrated the sensitisation of the Ln(iii) emission by the **tdt** ligand *via* an energy transfer mechanism. It is noteworthy that the luminescent ternary assembly formed was thermodynamically stable and retained its emission intensity and colour even after being kept in solution for one month at room temperature (Fig. S22, ESI†).

Further measurements on final assemblies, where the molar ratio *χ*_Eu_ of Eu(EDTA)·(H_2_O)_3_ was varied from 0 → 1 showed that white light emission could not be achieved under these particular conditions (Fig. S23, ESI†). However, the fact that the **tdt** ligand is built from two different chromophoric units, both capable of sensitising Ln(iii) emission, but possessing slightly different absorption spectra, the emission of the overall assembly can also be tuned by varying the excitation wavelength. The dependence of the emission spectra and corresponding CIE coordinates as a function of the excitation wavelength (Fig. S24 and Table S4, ESI†) showed that at *λ*_ex_ > 290 nm, *i.e.* when the light was almost uniquely absorbed by the tpy units, the ligand centred emission became the major contribution to the overall emission, and gave a distinct blue colour to the light emitted. When studying the photophysical properties of **tdt** in solution, the blue ligand fluorescence reached its maximum intensity at 4–5 μM and any increase in concentration resulted in a decrease of the fluorescence intensity. Therefore, the initial **tdt** concentration used for the formation of the luminescent assemblies is another parameter to be considered in achieving white light emission, as higher **tdt** concentrations should lessen the blue contribution to the overall emission. The same experiment was thus performed with [**tdt**] = 25 and 39 μM and the results were compared with the initial concentration of 12 μM. A relative decrease of the ligand emission was observed as expected with the increase in **tdt** concentration (Fig. S25 and Table S5, ESI†). Moreover, the calculated intensities ratio between the blue **tdt** emission and the red Eu(iii) emission demonstrated that only the intermediate concentration (25 μM) yielded values below unity at any excitation wavelengths. The weaker blue contribution to the emitted light compared to the other two concentrations is indeed fundamental to achieve white light emission as all the assemblies studied so far displayed blue emission that was too intense.

**Fig. 4:**
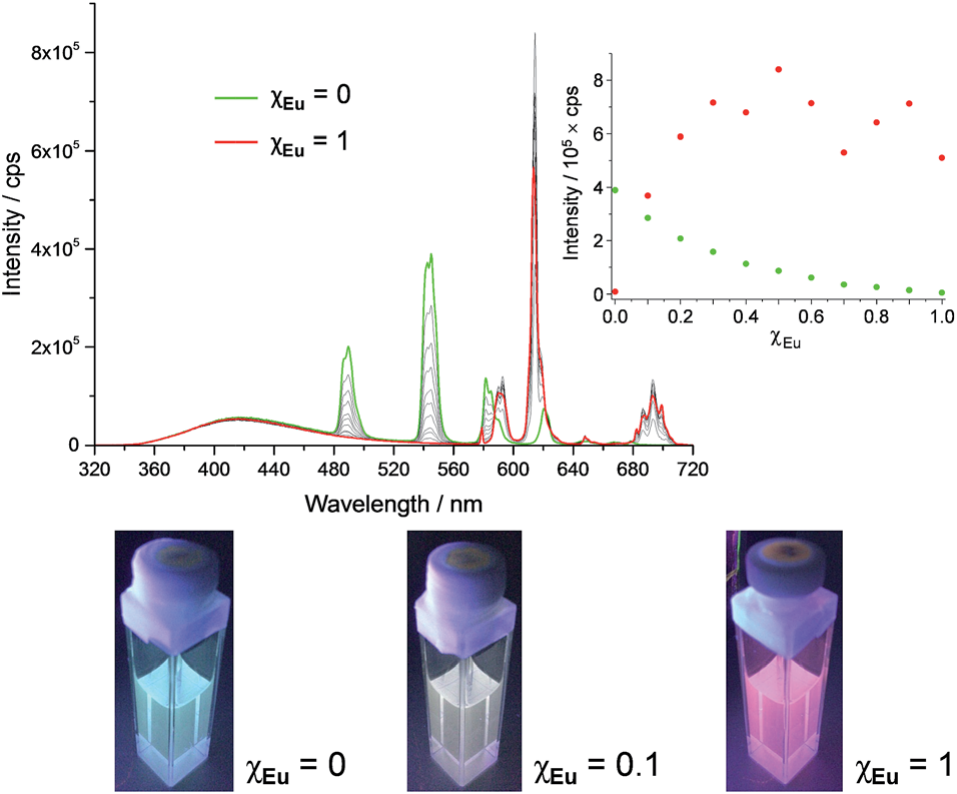
Fluorescence emission spectra of **tdt**[Eu(EDTA)]_*x*_[Tb(EDTA)]_3–*x*_ assemblies in methanol as a function of *χ*_Eu_ and corresponding pictures for *χ*_Eu_ = 0, 0.1 and 1; inset: experimental binding isotherms for the Eu(iii)- (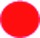) and Tb(iii)-centred (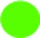) emission; [**tdt**] = 25 μM and *λ*_ex_ = 270 nm.

**Fig. 5:**
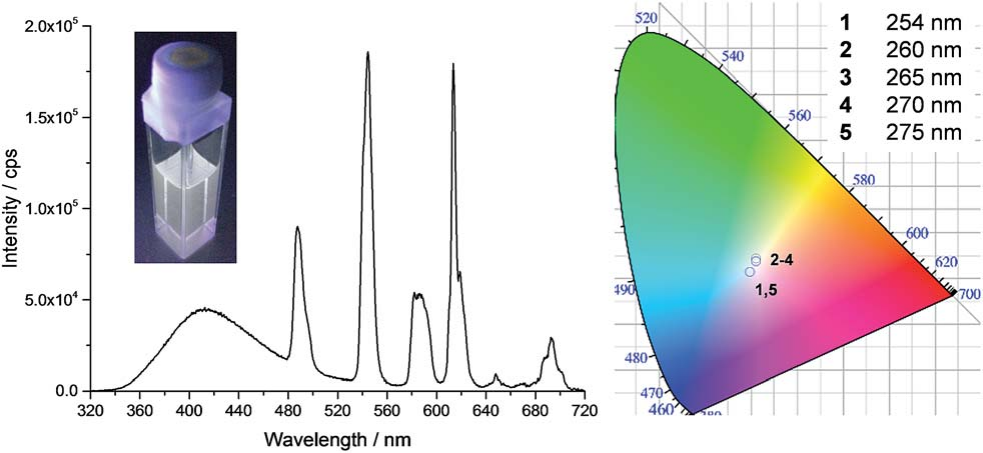
Fluorescence emission spectra (*λ*_ex_ = 270 nm) and CIE-1931 chromaticity diagram for **tdt**[Eu(EDTA)]_0.3_[Tb(EDTA)]_2.7_ in methanol as a function of the excitation wavelength; [**tdt**] = 25 μM and *χ*_Eu_ = 0.1. Inset shows the corresponding photograph obtained with *λ*_ex_ = 270 nm.

The series of experiments performed so far to obtain white light emission showed that to reach this goal, the optimal concentration of **tdt** had to be *ca.* 25 μM and that excitation wavelengths between 260 and 280 nm had to be used (Fig. S26, ESI†). Based on this knowledge, a series of heterometallic assemblies were prepared in solution, varying the molar ratio *χ*_Eu_ of Eu(EDTA)·(H_2_O)_3_ from 0 → 1. The resulting emission of the lanthanide self-assemblies was recorded, as shown in Fig. 4, and their CIE coordinates determined (Fig. S27, ESI†). Analysing the emission spectra first, particularly the intensities at 545 and 615 nm (the two most intense Tb(iii) and Eu(iii) transitions, respectively), it is clear that as soon as *χ*_Eu_ ≥ 0.2, the red Eu(iii) emission significantly overcomes the green emission of Tb(iii), giving a distinct pink-purple tint to the colour emitted, as observed above for **tdt**[Eu(EDTA)][Tb(EDTA)]_2_, for which *χ*_Eu_ = 0.33. Having established the optimal **tdt** concentration, the excitation wavelength and the molar ratio *χ*_Eu_, white light emission was successfully achieved for *χ*_Eu_ = 0.1 and *λ*_ex_ = 260–275 nm as demonstrated on Fig. 5 and Table S6 in the ESI.† However, once *λ*_ex_ > 280 nm, the same behaviour was observed as for previous attempts, namely a fast growing contribution of the blue ligand fluorescence with the increase in the excitation wavelength (Fig. S28, ESI†).

It is well-known that energy transfer often occurs between Ln(iii) ions in this type of multimetallic assembly.^23^ In order to investigate such possibility, we selected the **tdt**[Eu(EDTA)][Tb(EDTA)]_2_ and prepared two equivalent assemblies by replacing either Eu(iii) or Tb(iii) with Gd(iii), as the higher excited-state energy of the latter prevents its participation in any kind of energy transfer. As it is clearly shown in Fig. S29 in the ESI,† the Tb(iii) emission reached its maximum intensity in the Gd–Tb assembly, while the Eu(iii) emission was the most intense in the presence of Tb(iii), *i.e.* for the Eu–Tb assembly. The Eu(iii) emission intensity sustained a 2.5-fold increase compared to that observed for the Eu–Gd assembly. Concomitantly, the Tb(iii) emission displayed a *ca.* 20% decrease in intensity when comparing the Gd–Tb and Eu–Tb assemblies. These observations likely indicate that an energy transfer was taking place from the Tb(^5^D_4_) excited state to the Eu(^5^D_0_) one. The existence of such an energy transfer was further confirmed by a lengthening of the Eu(iii) excited state lifetime, alongside with a shortening of the Tb(iii) lifetime in **tdt**[Eu(EDTA)][Tb(EDTA)]_2_. In comparison, the lifetimes obtained for the Gd(iii)-based assemblies, Gd–Tb and Eu–Gd, matched well with the values determined for the homometallic assemblies (see Table S7 in the ESI†).

## Conclusions

In conclusion, we have developed a series of luminescent homo- and heterometallic lanthanide self-assembled complexes from the **tdt** ligand. The tpy and dpa units of the latter ensured high stability of the assemblies in solution, as well as provided suitable antennae for the sensitisation of the lanthanide emission. The homometallic assemblies displayed the characteristic green and red emission of Tb(iii) and Eu(iii), respectively, with a considerable blue ligand fluorescence attributed to the tpy units of **tdt**. White-light emission was successfully achieved by carefully tuning (i) the molar ratio of Eu(iii) and Tb(iii) within the assembly and thus the intensity of the red and green emission, (ii) the excitation wavelength, as the **tdt** ligand consists of two different chromophores, and (iii) the ligand concentration, which greatly affects the intensity of the blue emission within the overall self-assembled complexes. In addition to their application in white-light-emitting materials, these heterometallic assemblies demonstrate good potential for the development of ratiometric sensors, based on three colours and two time domains (ns range for the ligand blue fluorescence and ms range for the lanthanide emission). Moreover, their high solubility and stability in methanol make them suitable for incorporation into polymer matrices or for use as coatings on solid support; an endeavour that we are currently investigating.

## Experimental

### Materials and methods

All solvents and chemicals were purchased from commercial sources and used without further purification. Dichloromethane was freshly distilled under argon atmosphere prior to use. Water was purified using a Millipore Milli-Q water purification system. Triethylamine, ethylenediaminetetraacetic acid (EDTA), 4′-chloro-[2,2′:6′,2′′]terpyridine (4′-chloroterpyridine), 1,3-diamino propane, 2,6-pyridinedicarboxylic acid (dipicolinic acid, dpa) and Ln(NO_3_)_3_·6H_2_O (Ln(iii) = La, Eu, Gd, Tb and Yb) were purchased from Sigma-Aldrich. Stock solutions of lanthanides were prepared just before use in MeOH (HPLC grade) from the corresponding nitrate salts. Exact concentrations of the solutions were determined by complexometric titrations using a standardized Na_2_H_2_EDTA solution in urotropine buffered medium and xylenol orange as the indicator. Deuterated solvent used for NMR analysis (DMSO-d_6_) was purchased from Apollo Scientific. The ^1^H and ^13^C NMR spectra were recorded using a Bruker AV-600 instrument operating at 600.1 MHz for ^1^H NMR and 150.2 MHz for ^13^C NMR. Chemical shifts are reported in ppm using deuterated solvents as internal standards. All NMR data acquisition were carried out at 293 K. Melting points were determined using an Electrothermal IA9000 digital melting point apparatus. Mid-infrared spectra were recorded using a Perkin-Elmer Spectrum One FT-IR spectrometer equipped with a universal attenuated total reflection (ATR) sampling accessory. Elemental analysis was conducted at the Microanalytical Laboratory, School of Chemistry and Chemical Biology, University College Dublin.

### General synthetic procedure

#### Pyridine-2,6-dicarbonyl dichloride (**5**)

Pyridine-2,6-dicarbonyl dichloride (**5**) has been synthesized following a literature procedure commonly used in our research group.^18*b*^

#### *N*-[2,2′:6′,2′′]Terpyridin-4′-yl-propane-1,3-diamine (**4**)

The synthesis of the amino derivative of tpy was performed following the same procedure as the one used recently published by our group.^17*a*^

#### *N*_2_,*N*_6_-Bis(3-(2,6-di(pyridin-2-yl)pyridin-4-ylamino)propyl)pyridine-2,6-dicarboxamide (**tdt**)

To a solution of *N*-[2,2′:6′,2′′]terpyridin-4′-yl-propane-1,3-diamine (**4**, 0.075 g, 2.46 × 10^–4^ mol) and triethylamine (0.035 mL, 2.51 × 10^–4^ mol) in dichloromethane (3 mL) at 0 °C under an atmosphere of argon was added slowly 2,6-pyridinedicarbonyl dichloride (**5**, 0.025 g, 1.23 × 10^–4^ mol). The reaction was then stirred at room temperature overnight resulting in the formation of a white precipitate which was filtered and washed with water. The obtained white powder was dried under vacuum and used without any further purification (0.062 g, yield = 67.4%). Mp 145 °C; ^1^H NMR (600 MHz, (CD_3_)_2_SO, *δ*_H_) 8.78 (s, 2H, NH), 8.67 (d, *J* = 4.68 Hz, 4H, CH_tpy_), 8.56 (d, *J* = 7.92, 4H, CH_tpy_), 7.96 (td, ^3^ *J*_H–H_ = 7.68 Hz, ^4^ *J*_H–H_ = 1.74 Hz, 4H, CH_tpy_), 7.90 (d, *J* = 7.56 Hz, 2H, CH_py_), 7.76 (t, *J* = 7.56 Hz, 1H, CH_py_), 7.68 (s, 4H, CH_tpy_), 7.44 (td, ^3^ *J*_H–H_ = 6.11 Hz, ^4^ *J*_H–H_ = 0.96 Hz, 4H, CH_tpy_), 7.02 (s, 2H, NH), 3.35 (t, *J* = 6.66 Hz, 4H, CH_2_), 2.94 (t, *J* = 7.14 Hz, 4H, CH_2_), 1.97 (m, 4H, CH_2_); ^13^C NMR (150 MHz, (CD_3_)_2_SO, *δ*_C_) 168.6, 155.9, 155.4, 155.0, 154.7, 148.9, 137.0, 136.4, 123.8, 123.4, 120.6, 103.8, 39.3, 36.5, 26.7; IR *ν*_max_ (cm^–1^): 3282, 3157, 3060, 2946, 2859, 1601, 1582, 1562, 1466, 1439, 1408, 1362, 1257, 1223, 1147, 1092, 1046, 984, 912, 847, 790, 720, 653; anal. calc. for C_43_H_39_N_11_O_2_·2HCl·1.5H_2_O, %: C 61.4, H 5.3, N 18.3; found, %: C 61.5, H 5.6, N 18.3.

### Photophysical measurements

Unless otherwise stated, all measurements were performed at 298 K in methanol solution (HPLC grade). UV-visible absorption spectra were measured in 1 cm quartz cuvettes on a Varian Cary 50 spectrophotometer. Baseline correction was applied for all spectra. Emission (fluorescence, phosphorescence and excitation) spectra and lifetimes were recorded either on a Varian Cary Eclipse Fluorimeter or a Fluorolog FL 3-22 spectrophotometer from Horiba Jobin Yvon. Quartz cells with a 1 cm path length from Hellma were used for these measurements. Phosphorescence lifetimes of the Tb(^5^D_4_) and Eu(^5^D_0_) excited states were measured at 298 K on the Varian Cary Eclipse Fluorimeter. They are averages of three independent measurements, where the emission intensity at 545 and 616 nm, which corresponds to the maxima of the Tb(iii) ^5^D_4_ → ^7^F_5_ and Eu(iii) ^5^D_0_ → ^7^F_2_ transitions, respectively were monitored as a function of time enforcing a 0.1 ms initial delay. The resulting exponential decay curves were then analysed using Origin 7.5®.

Fluorescence lifetime measurements were performed with a Horiba Jobin Yvon Fluorolog FL 3-22 equipped with a FluoroHub v2.0 single photon controller using the time-correlated single photon counting method (TCSPC), run in reverse mode. The sample solutions were excited at 294 nm with a pulsed nanosecond light-emitting diode (NanoLED®). The time distribution of the lamp pulse (<1.0 ns), also called the instrument response function, was recorded prior to lifetime measurements in a separate experiment using a scatter solution, in this case a solution of silica nanoparticles (Ludox® from Aldrich). All the measurements were performed at 298 K. The decays were analysed using IBH DAS6 software and the data fitted as a sum of exponentials, employing a nonlinear least-squares error minimization analysis:
1

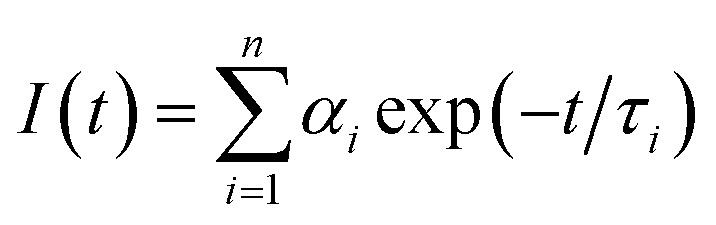




The errors are taken as two standard deviations. The goodness of the fit was assessed by the chi-squared value as well as the symmetric distribution of the residuals about the zero axes. The average lifetime 〈*τ*〉 is given by

2 〈*τ*〉 = ∑*α*_*i*_
*τ*_*i*_^2^/∑*α*_*i*_
*τ*_*i*_ = ∑*f*_*i*_
*τ*_*i*_

where the fractional contribution of the lifetime *i* is defined as 
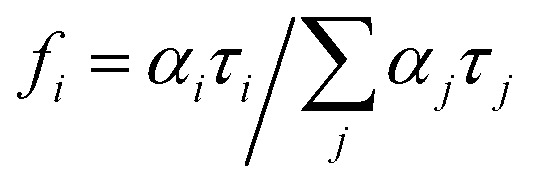
.

### Spectrophotometric titrations and binding constants

In a typical spectrophotometric titration, 2.7 mL of ligand **tdt** (5–10 μM) in MeOH was titrated with a solution of either Ln(NO_3_)_3_·6H_2_O (Ln(iii) = La, Eu, Gd, Tb, Yb) or Ln(EDTA)·(H_2_O)_3_ (Ln(iii) = Eu, Gd, Tb). In the latter case, stock solutions were prepared by adding 1 equivalent of Ln(NO_3_)_3_·6H_2_O to a 5 mM solution of EDTA and let to stir and equilibrate overnight to ensure complete formation of the 1 : 1 (Ln : L, where Ln = lanthanide and L = EDTA) complexes, Ln(EDTA)·(H_2_O)_3_. The UV-visible data were then treated and fitted using the non-linear least squares regression analysis program, SPECFIT® to determine the stoichiometry of the metal species formed in solution as well as their corresponding stability constants. Residuals calculated for UV-visible data are equal to 1.6 × 10^–3^ and 2.8 × 10^–3^ for Eu(EDTA)·(H_2_O)_3_ and Tb(EDTA)·(H_2_O)_3_, respectively.

### White light experiments and solution stability of heterometallic assemblies

Solutions containing different stoichiometric ratios of **tdt** : Eu(EDTA) : Tb(EDTA) were prepared in individual sample vials and kept at room temperature. To ensure that equilibrium has been reached the solution were left equilibrating for 24 hours before carrying out any measurements. The initial concentration of **tdt** in these samples was varied from 10 to 39 μM. The stability of the heterometallic assemblies in solution was assessed by measuring the emission and excitation spectra over a period of 30 days. The solutions for the measurements on the final **tdt**[Eu(EDTA)]_*x*_[Tb(EDTA)]_3–*x*_ assemblies, where the molar ratio *χ*_Ln_ was changed from 0 → 1, were prepared as follow: the total number of moles of Ln(EDTA)·(H_2_O)_3_ was kept constant at a value that is three times the one of the **tdt** ligand, so that all the three binding sites will be occupied. In these conditions, the molar ratio of Eu(EDTA)·(H_2_O)_3_·(*χ*_Eu_) was varied ensuring that (*χ*_Eu_ + *χ*_Tb_ = 1) for each sample prepared. Consequently, for a *χ*_Eu_ = 0.33, the final assembly formed has an overall formula corresponding to **tdt**[Eu(EDTA)][Tb(EDTA)]_2_. Spectral analyses to obtain CIE coordinates were performed using a CIE coordinate calculator running on Mathlab®.
