# Supplementary material for: White-light emission from discrete heterometallic lanthanide-directed self-assembled complexes in solution
Source: Chem Sci. 2017 Mar 6;8(5):3419–26. doi: 10.1039/c7sc00739f (PMC5417009; doi:10.1039/c7sc00739f)
Supplement: Supplementary file 1 [file SC-008-C7SC00739F-s001.pdf]

Electronic Supplementary Material (ESI) for Chemical Science.
This journal is © The Royal Society of Chemistry 2017

Journal Name

ARTICLE

Electronic Supplementary Information for

White-Light Emission from Discrete Heterometallic Lanthanide-Directed Self-Assembled Complexes in Solution

Oxana Kotova†, Steve Comby†*, Christophe Lincheneau and Thorfinnur Gunnlaugsson*

Table of Contents

Table of contents

Part I. NMR characterisation of ligand tdt p. S2-S4

Part II. Physicochemical characterization and Luminescence studies p. S5-S16

This journal is © The Royal Society of Chemistry 20xx

Part I.

A

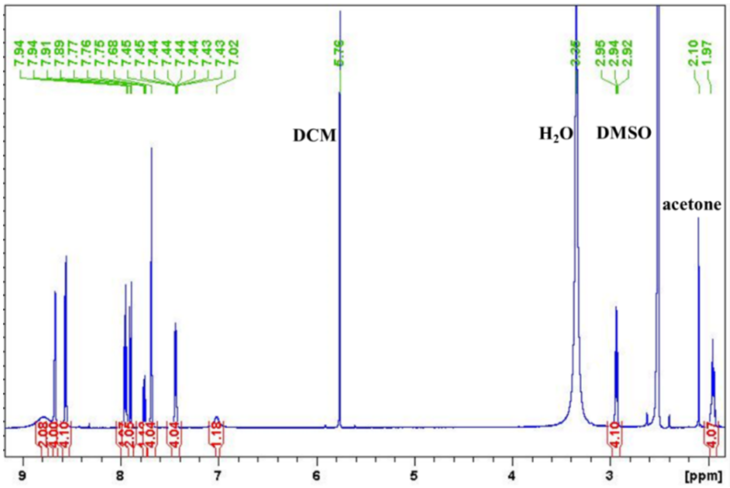

B

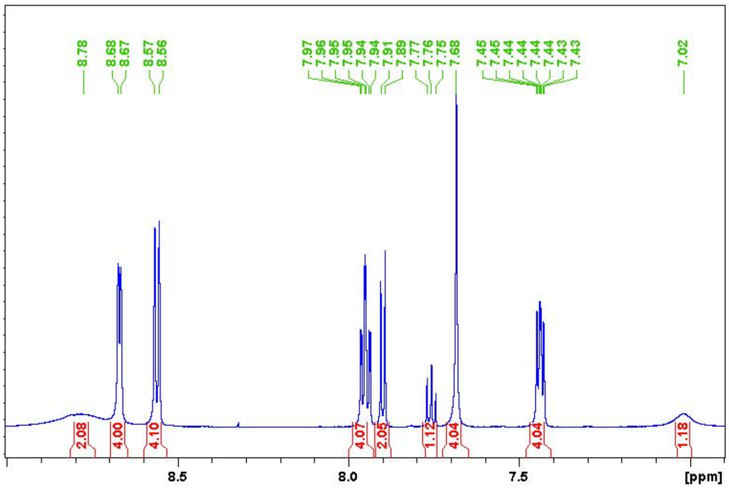

Fig. S1 ^{1}H NMR of ligand **tdt** (600 MHz, $(CD_3)_2SO$); (A) full spectrum, (B) focus on the aromatic region.

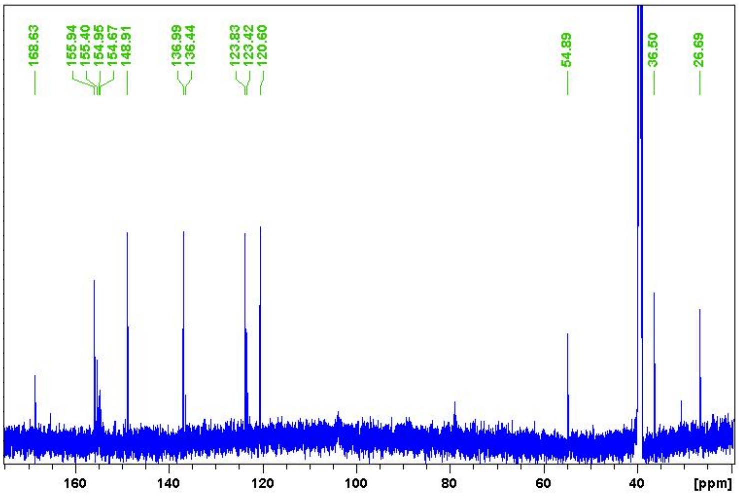

 This journal is © The Royal Society of Chemistry 20xx

Please do not adjust margins

Fig. S2 ^{13}C NMR of ligand **tdt** (150 MHz, $(CD_3)_2SO$).

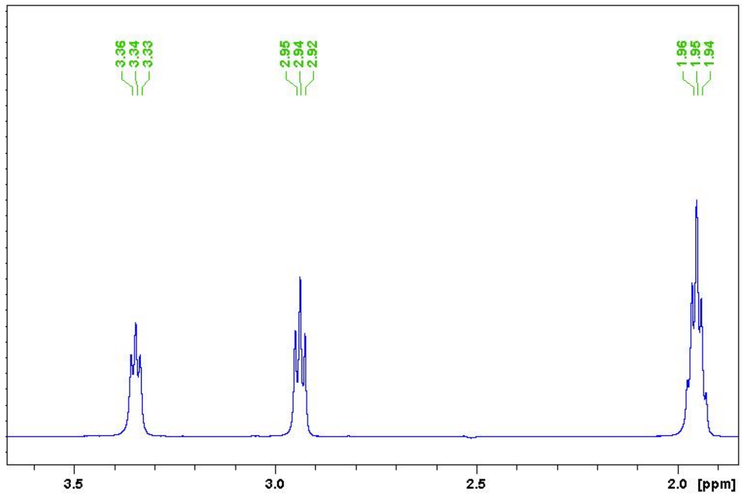

Fig. S3 1D TOCSY spectrum of ligand **tdt** with irradiation of CH_2 in $(CD_3)_2SO$.

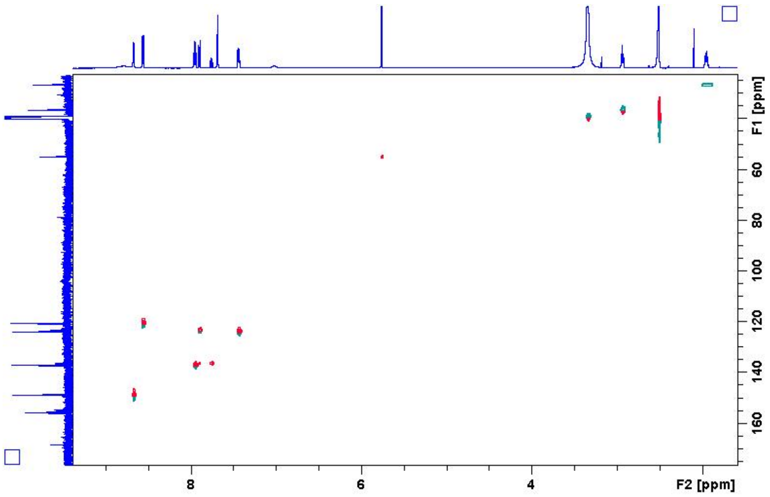

Fig. S4 ^{1}H–^{13}C HSQC experiment of ligand **tdt** in $(CD_3)_2SO$.

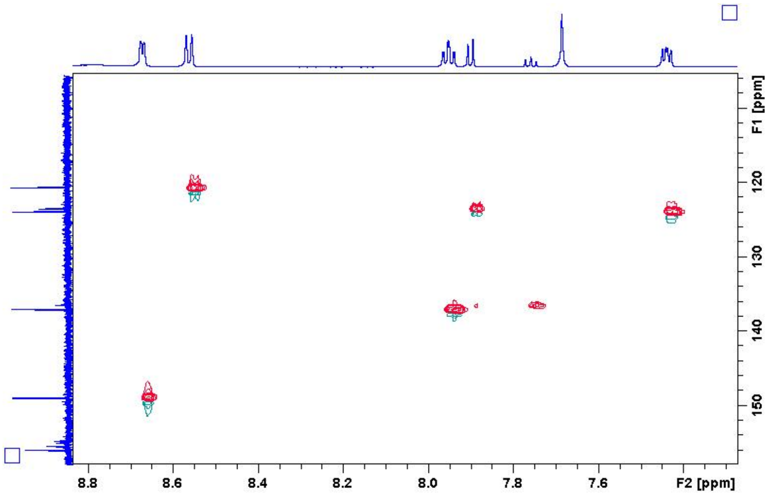

Please do not adjust margins

Please do not adjust margins

Fig. S5 ^{1}H–^{13}C HSQC experiment of ligand **tdt** in $(CD_3)_2SO$ showing zoomed areas.

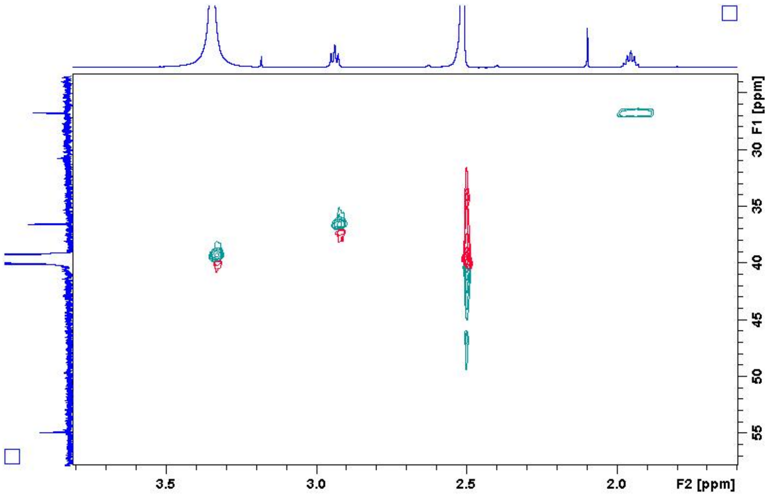

Fig. S6 ^{1}H–^{13}C HSQC experiment of ligand **tdt** in $(CD_3)_2SO$ showing zoomed areas.

This journal is © The Royal Society of Chemistry 20xx

Please do not adjust margins

Part II.

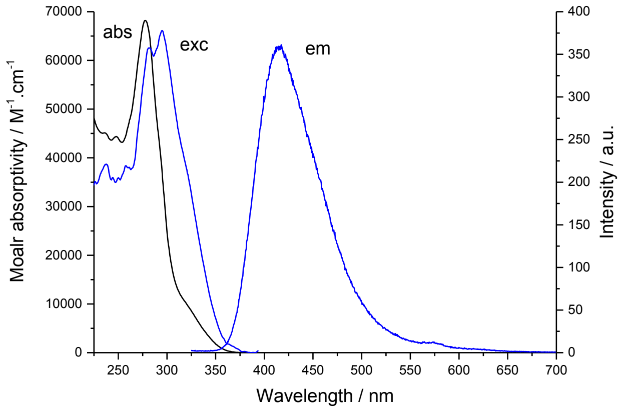

Fig. S7 Absorption, emission (λ_{ex} = 280 nm) and excitation (λ_{an} = 415 nm) spectra of **tdt** in methanol; [**tdt**] = 5 μM.

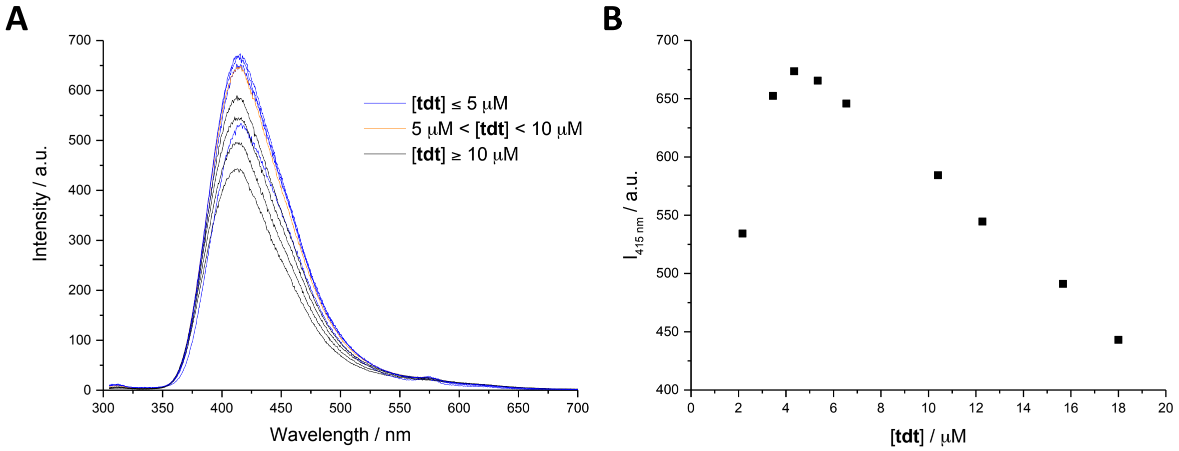

Fig. S8 Fluorescence emission spectra (A) and corresponding changes in the fluorescence intensities at 415 nm (B) as a function of **tdt** concentration in methanol; [**tdt**] = 2-18 μM.

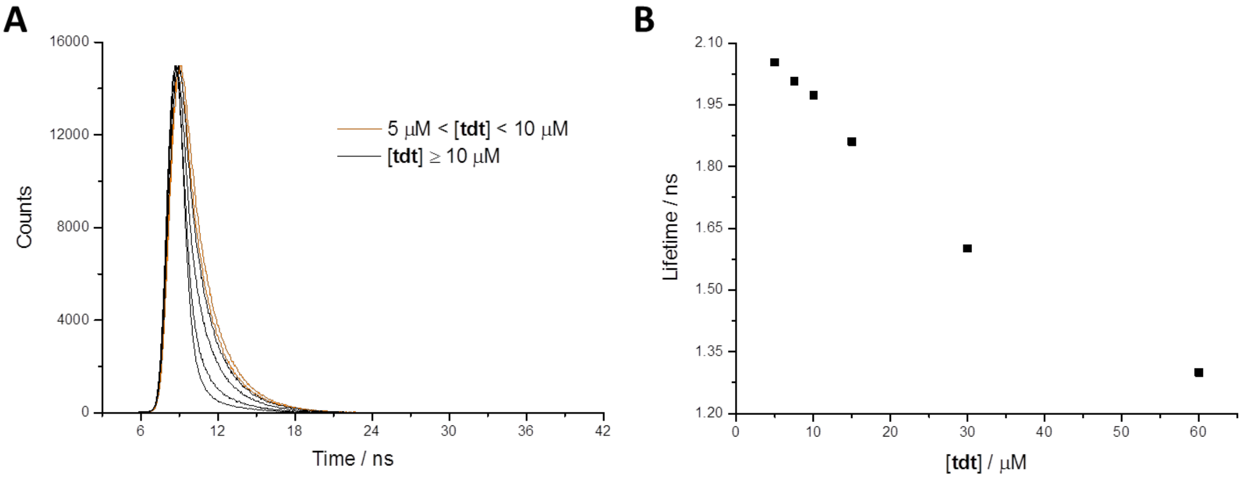

Fig. S9 Fluorescence emission decays (A) and corresponding lifetime values (B) as a function of **tdt** concentration in methanol; [**tdt**] = 5-60 μM.

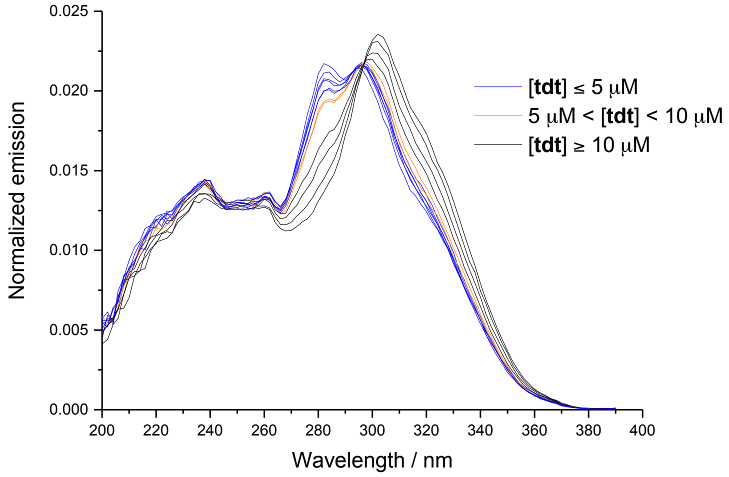

Fig. S10 Fluorescence excitation spectra as a function of **tdt** concentration in methanol; [**tdt**] = 2-18 μM.

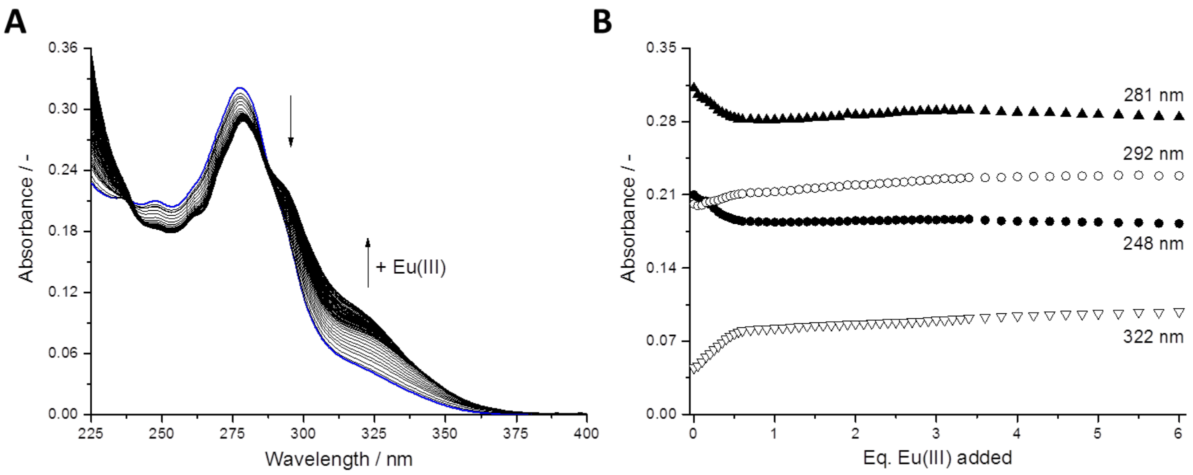

Fig. S11 (A) Absorption spectra and (B) experimental binding isotherms at various wavelengths for the UV-visible titration of **tdt** (5 μM) with $Eu(NO_3)_3$ in methanol at 298 K.

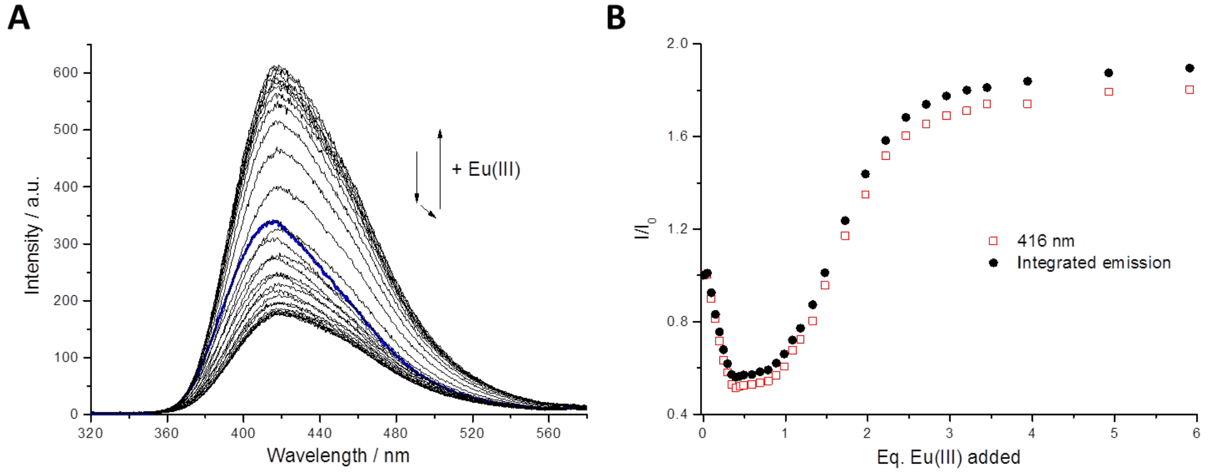

Fig. S12 (A) Changes in the ligand-centered fluorescence emission of **tdt** (5 μM) upon addition of $Eu(NO_3)_3$ in methanol at room temperature, (B) experimental binding isotherms of the fluorescence intensity and integrated emission as a function of the equivalents of Eu(III) added.

This journal is © The Royal Society of Chemistry 20xx

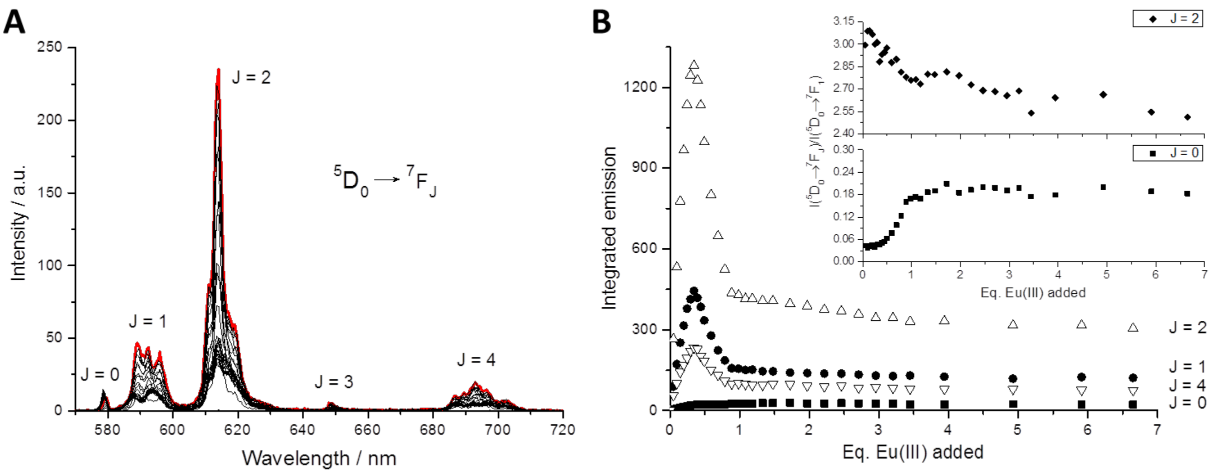

Fig. S13 (A) Changes in the Eu(III)-centred phosphorescence emission upon addition of $Eu(NO_3)_3$ to a solution of **tdt** (5 μM) in methanol at 298 K. (B) Experimental binding isotherms of the phosphorescence integrated emission over the 570-720 nm range as a function of the equivalents of Eu(III) added; Inset shows the intensities of the $^5D_0 \rightarrow {}^7F_J$ transitions (J = 0, 2), relative to the magnetic dipole $^5D_0 \rightarrow {}^7F_1$ transition.

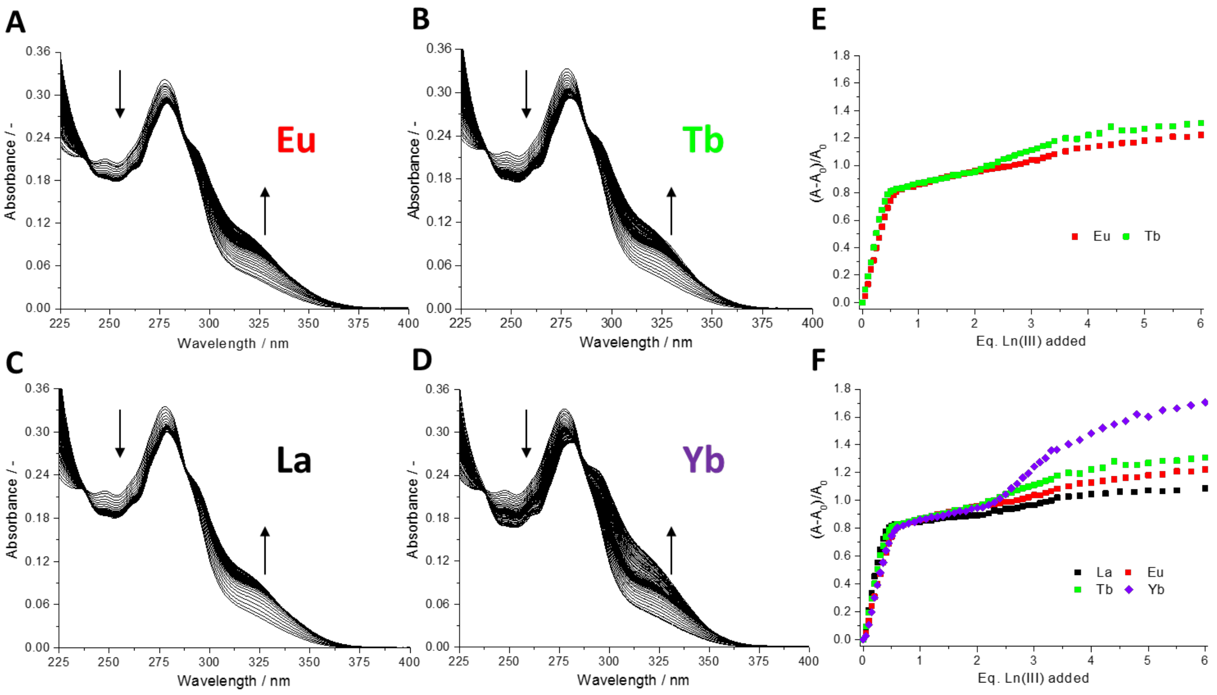

Fig. S14 (A-D) Changes in the UV-visible absorption spectrum of **tdt** (5 μM) upon addition of $Ln(NO_3)_3$ (Ln = La, Eu, Tb and Yb) in methanol at 298 K. (E-F) Plots of $(A-A_0)/A_0$ vs. the equivalents of $Ln(NO_3)_3$ added to the **tdt** solution, where A_0 and A denotes the absorption of **tdt** at 322 nm before and after addition of $Ln(NO_3)_3$,respectively.

Please do not adjust margins

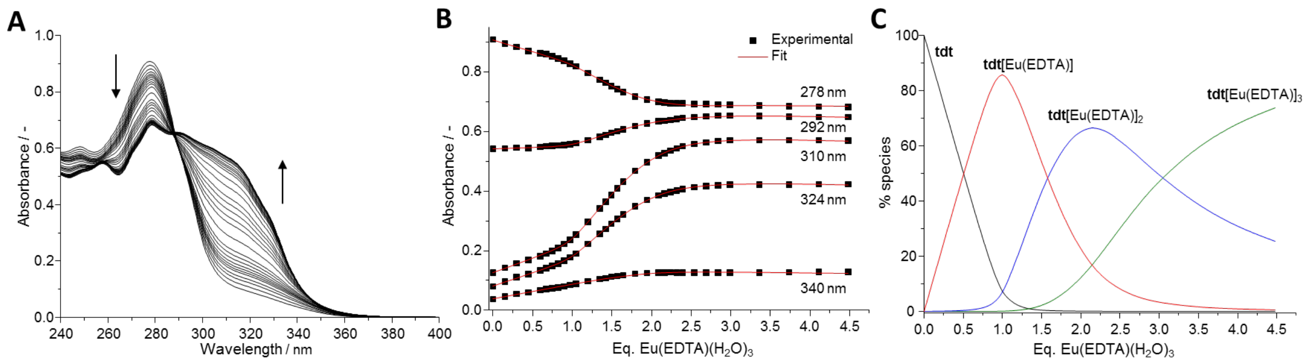

Fig. S15 (A) Absorption spectra, (B) experimental binding isotherms with corresponding fits (—) and (C) speciation-distribution diagram for the UV-visible titration of tdt (12 μM) with $Eu(EDTA)(H_2O)_3$ in methanol at 298 K.

Table S1 Stoichiometries and cumulative binding constants of the species present in solution as determined from the UV-visible titrations of **tdt** with $Ln(EDTA)(H_2O)_3$; Ln = Eu, Tb.

	$\log\beta_{11}$	$\log\beta_{12}$	$\log\beta_{13}$
**tdt**:$Ln(EDTA)(H_2O)_3$	1:1	1:2	1:3
Ln = Eu	8.5 ± 0.2	14.8 ± 0.3	20.0 ± 0.3
Ln = Tb	9.6 ± 0.2	17.6 ± 0.3	23.7 ± 0.4

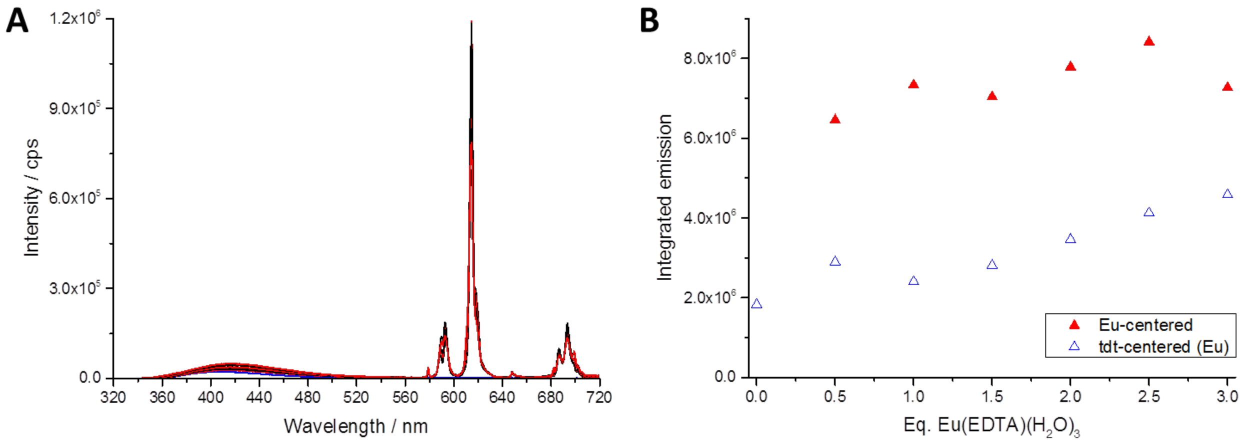

Fig. S16 (A) Fluorescence emission spectra of the homometallic assemblies formed in solution between **tdt** and $Eu(EDTA)(H_2O)_3$, (B) experimental binding isotherms for the integrated ligand- and Eu(III)-centred fluorescence emission as a function of the equivalents of $Eu(EDTA)(H_2O)_3$ added.

This journal is © The Royal Society of Chemistry 20xx

Please do not adjust margins

Please do not adjust margins

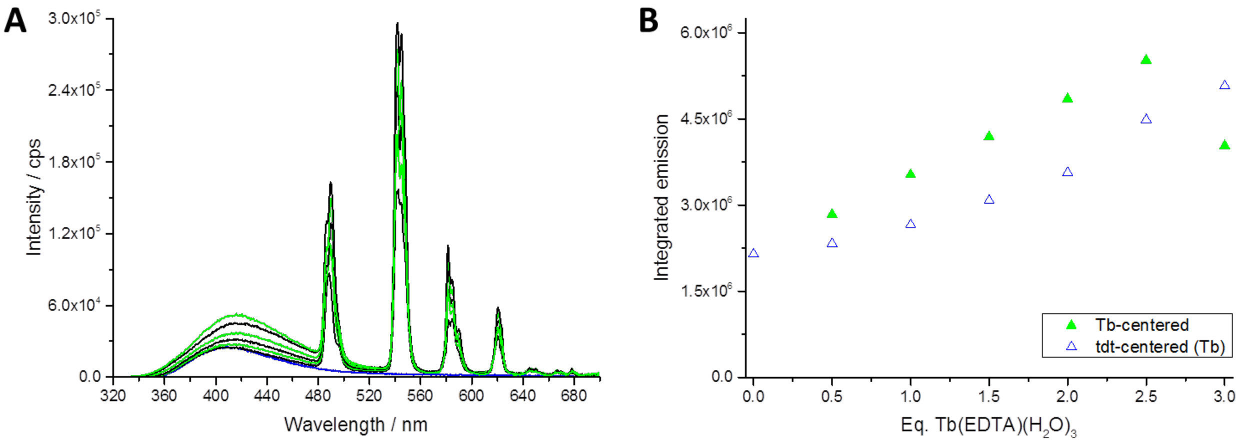

Fig. S17 (A) Fluorescence emission spectra of the homometallic assemblies formed in solution between **tdt** and Tb(EDTA)(H_2O)$_3$, (B) experimental binding isotherms for the integrated ligand- and Tb(III)-centred fluorescence emission as a function of the equivalents of Tb(EDTA)(H_2O)$_3$ added.

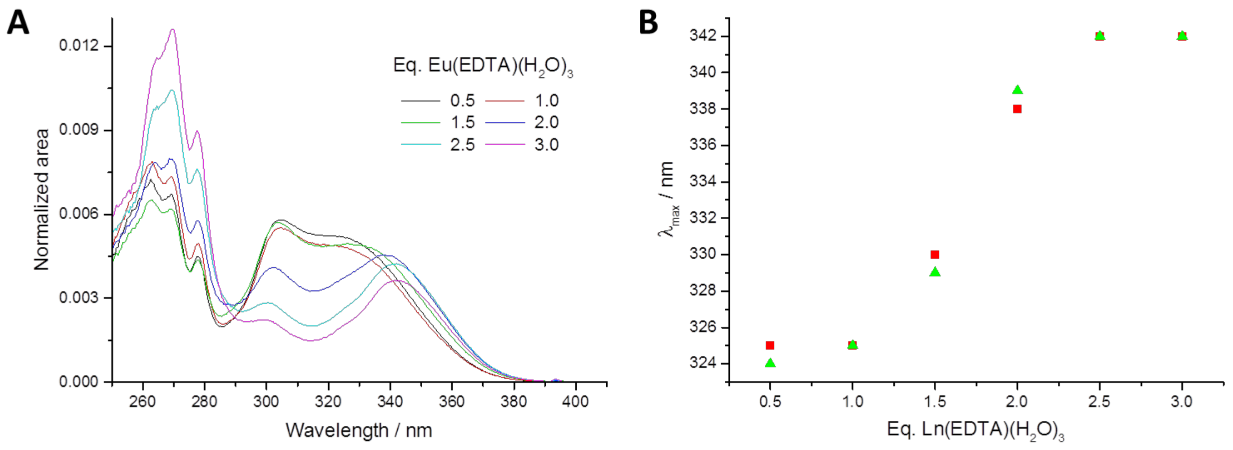

Fig. S18 (A) Excitation spectra (λ_{an} = 615 nm) of the Eu(III)-based homometallic assemblies as a function of the equivalents of Eu(EDTA)(H_2O)$_3$ added, (B) shift observed in the maxima of the lowest energy band upon formation of the Eu(III)- (red circles) and Tb(III)-based assemblies (green triangles).

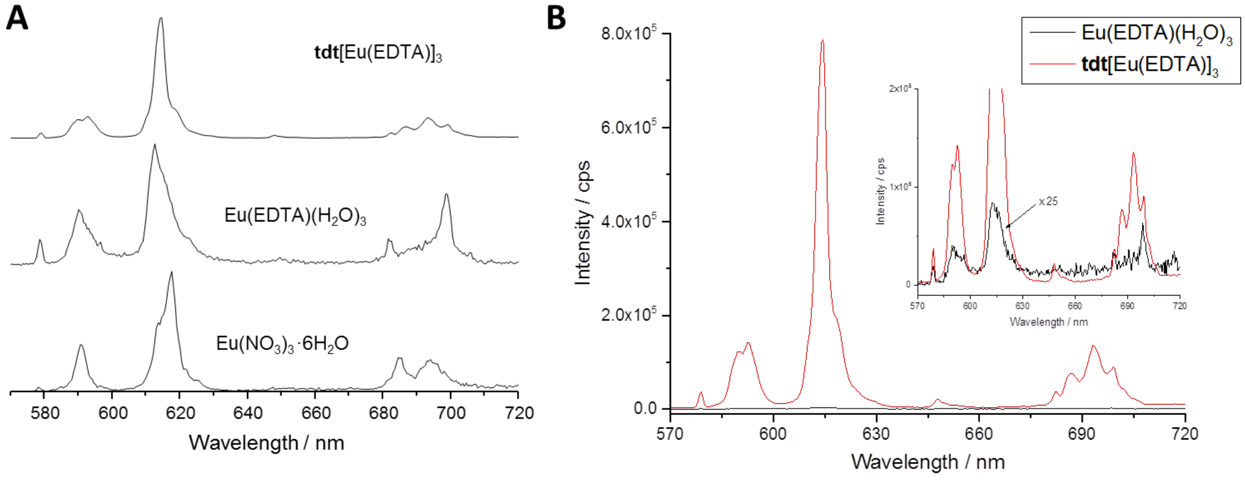

Fig. S19 (A) Overall shape and relative intensities for the Eu(III)-centred emission of the ternary assembly **tdt**[Eu(EDTA)]$_3$, Eu(EDTA)(H_2O)$_3$ and Eu(NO_3)$_3$·6H_2O measured using identical experimental parameters, (B) comparison of the Eu(III)-centred emission intensity between **tdt**[Eu(EDTA)]$_3$ and Eu(EDTA)(H_2O)$_3$, measured using the same experimental parameters and a total Eu(EDTA)(H_2O)$_3$ concentration of 70 μM in both cases.

This journal is © The Royal Society of Chemistry 20xx

Please do not adjust margins

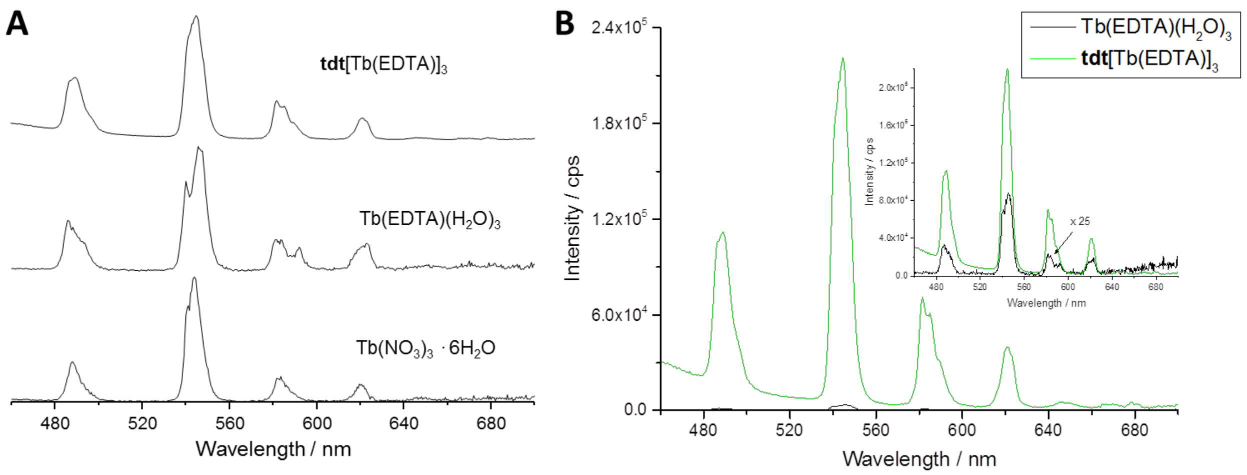

Fig. S20 (A) Overall shape and relative intensities for the Tb(III)-centred emission of the ternary assembly **tdt**[Tb(EDTA)]$_3$, Tb(EDTA)(H_2O)$_3$ and Tb(NO_3)$_3$·6H_2O measured using identical experimental parameters, (B) comparison of the Tb(III)-centred emission intensity between **tdt**[Tb(EDTA)]$_3$ and Tb(EDTA)(H_2O)$_3$, measured using the same experimental parameters and a total Tb(EDTA)(H_2O)$_3$ concentration of 70 µM in both cases.

Table S2 Intensities of the $^5D_0 \rightarrow ^7F_J$ transitions, relative to the magnetic dipole $^5D_0 \rightarrow ^7F_1$ transition and identified crystal-field sublevels (cm^{-1}) of the ^7F_J manifold ($^7F_0$ is taken as the origin) of **tdt**[Eu(EDTA)]$_3$, Eu(EDTA)(H_2O)$_3$ and Eu(NO_3)$_3$·6H_2O measured at 298 K.

Cpd	$^5D_0 \rightarrow ^7F_0$	$^5D_0 \rightarrow ^7F_1$	$^5D_0 \rightarrow ^7F_2$	$^5D_0 \rightarrow ^7F_3$	$^5D_0 \rightarrow ^7F_4$
Relative intensities					
**tdt**[Eu(EDTA)]$_3$	0.05	1.00	4.17	0.08	1.46
Eu(EDTA)(H_2O)$_3$	0.12	1.00	2.94	0.11	1.83
Eu(NO_3)$_3$·6H_2O	0.02	1.00	3.83	0.10	2.05
Crystal-Field Sublevels					
**tdt**[Eu(EDTA)]$_3$	0*	322, 408	905, 998, 1090, 1220	1839, 1875, 1934	2608, 2715, 2841, 2965, 3026, 3077
Eu(EDTA)(H_2O)$_3$	0*	337, 423	946, 1026, 1209	1901, 1984	2613, 2835, 2970, 3082
Eu(NO_3)$_3$·6H_2O	0*	366	1014, 1107, 1211	1987	2628, 2702, 2840, 2892, 2974, 3056

* $E(^5D_0 \rightarrow ^7F_0)$ = 17271, 17286 and 17301 cm^{-1} for **tdt**-Eu(EDTA)$_3$, Eu(EDTA)(H_2O)$_3$ and Eu(NO_3)$_3$·6H_2O, respectively

Table S3. Intensities of the $^5D_4 \rightarrow ^7F_J$ transitions, relative to the $^5D_4 \rightarrow ^7F_5$ transition and identified crystal-field sublevels (cm^{-1}) of the ^7F_J manifold ($^7F_6$ is taken as the origin) of **tdt**[Tb(EDTA)]$_3$, Tb(EDTA)(H_2O)$_3$ and Tb(NO_3)$_3$·6H_2O measured at 298 K.

Cpd	$^5D_4 \rightarrow ^7F_6$	$^5D_4 \rightarrow ^7F_5$	$^5D_4 \rightarrow ^7F_4$	$^5D_4 \rightarrow ^7F_3$	$^5D_4 \rightarrow ^7F_{2,1,0}$
Relative intensities					
**tdt**[Tb(EDTA)]$_3$	0.45	1.00	0.31	0.14	< 0.05
Tb(EDTA)(H_2O)$_3$	0.36	1.00	0.26	0.17	< 0.1
Tb(NO_3)$_3$·6H_2O	0.31	1.00	0.21	0.12	< 0.1
Crystal-Field Sublevels					
**tdt**[Tb(EDTA)]$_3$	0*, 105, 394	2105, 2206, 2307	3358, 3461, 3577	4452, 4504	5099, 5585, 5827
Tb(EDTA)(H_2O)$_3$	0*, 210	2058, 2261	3394, 3482, 3684	4395, 4525	5215, 5651, 5870
Tb(NO_3)$_3$·6H_2O	0*, 167	2008, 2109	3339, 3456	4363	5060, 5544, 5786

* $E(^5D_4 \rightarrow ^7F_6)$ = 20555, 20576 and 20492 cm^{-1} for **tdt**[Tb(EDTA)]$_3$, Tb(EDTA)(H_2O)$_3$ and Tb(NO_3)$_3$·6H_2O, respectively

 This journal is © The Royal Society of Chemistry 20xx

Please do not adjust margins

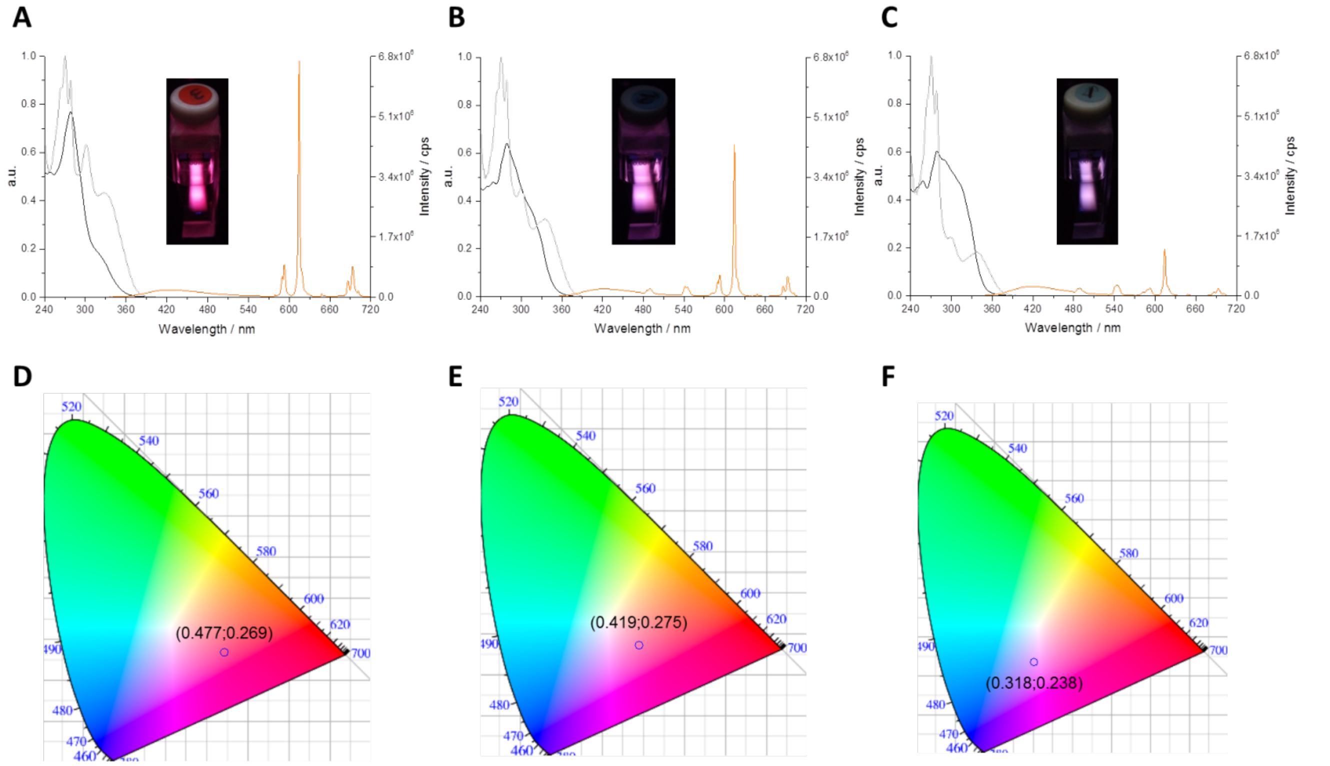

Fig. S21 (*Top*) UV-visible absorption, fluorescence emission and excitation spectra of **tdt** (12 µM) in methanol in the presence of (A) 1eq. $Eu(EDTA)(H_2O)_3$, (B) 1eq. $Eu(EDTA)(H_2O)_3$ + 1eq. $Tb(EDTA)(H_2O)_3$, and (C) 1eq. $Eu(EDTA)(H_2O)_3$ + 2eq. $Tb(EDTA)(H_2O)_3$; λ_{ex} = 280 nm, λ_{an} = 616 nm. Inset shows the pictures of the solutions A, B and C under 280 nm irradiation; (*Bottom*) (D-F) corresponding CIE-1931 chromaticity diagrams for the assemblies A-C.

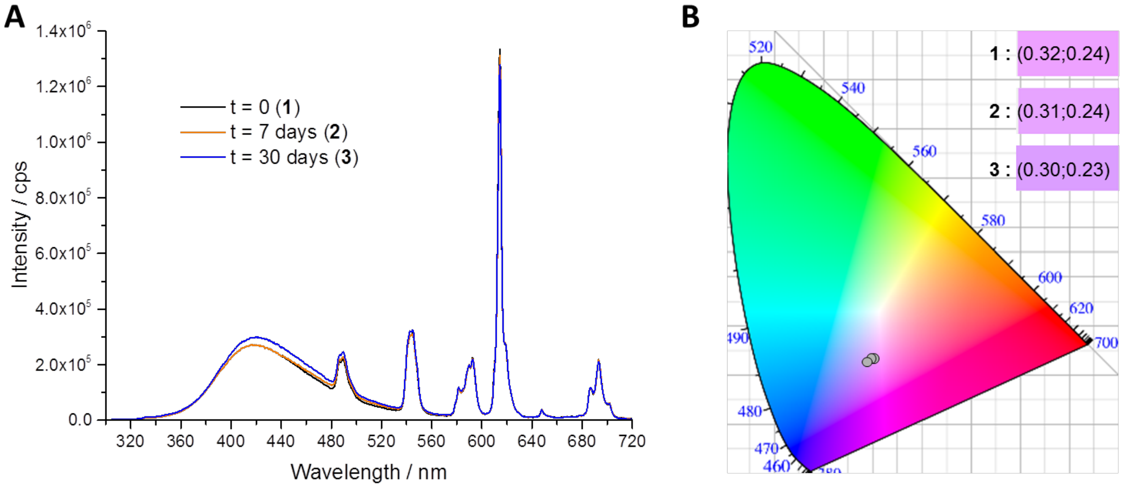

Fig. S22 (A) Fluorescence emission spectra and (B) corresponding CIE-1931 chromaticity diagram of a **tdt** solution (12 µM) in methanol in the presence of 1eq. $Eu(EDTA)(H_2O)_3$ + 2eq. $Tb(EDTA)(H_2O)_3$, measured at time t=0, 7 and 30 days; λ_{ex} = 280 nm.

Please do not adjust margins

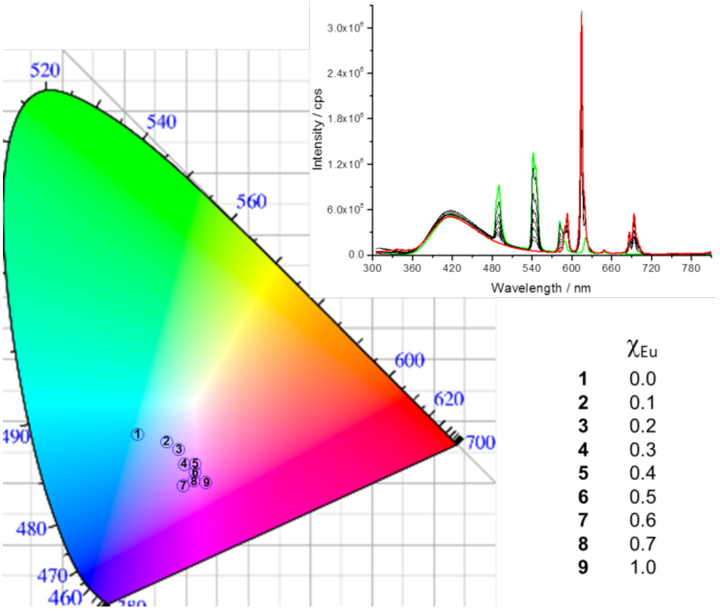

Fig. S23 Fluorescence emission spectra and corresponding CIE-1931 chromaticity diagram for the various **tdt**[Eu(EDTA)]$_x$[Tb(EDTA)]$_{3-x}$ assemblies in methanol as a function of χ_{Eu}, the molar ratio of Eu(EDTA)(H_2O)$_3$; [**tdt**] = 12 µM and λ_{ex} = 280 nm.

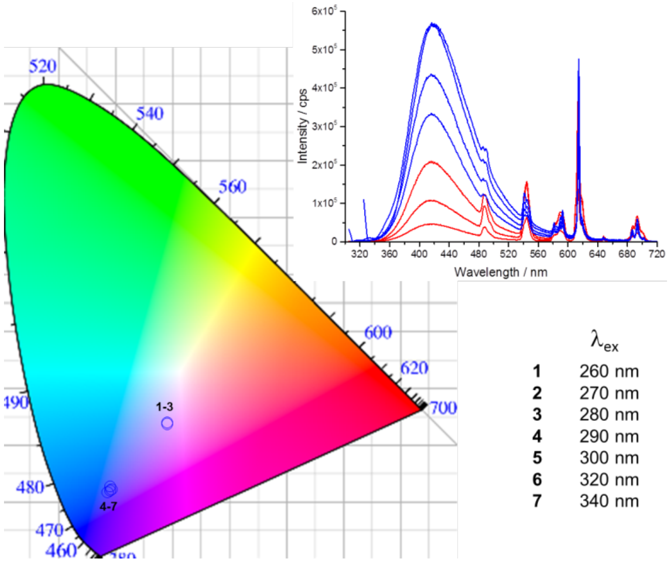

Fig. S24 Fluorescence emission spectra and corresponding CIE-1931 chromaticity diagram for the **tdt**[Eu(EDTA)] [Tb(EDTA)]$_2$ assembly in methanol as a function of the excitation wavelength, λ_{ex} = 260→340 nm; [**tdt**] = 12 µM, T = 298 K.

This journal is © The Royal Society of Chemistry 20xx

Please do not adjust margins

Table S4 CIE (x,y) coordinates and pictures of the light emitted by **tdt**[Eu(EDTA)][Tb(EDTA)]$_2$ in MeOH as a function of the excitation wavelength (λ_{ex} = 260→340 nm); [**tdt**] = 12 μM, T = 298 K.

λ_{ex} / nm	260	270	280	290	300	320	340
CIE (x,y)	(0.303,0.241)	(0.305,0.243)	(0.302,0.232)	(0.195,0.127)	(0.190,0.123)	(0.186,0.120)	(0.190,0.131)
Picture	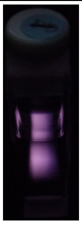	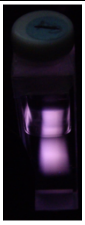	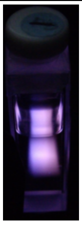	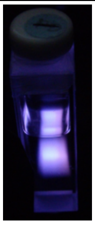	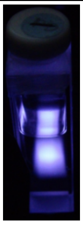	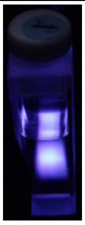	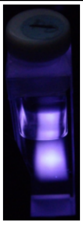

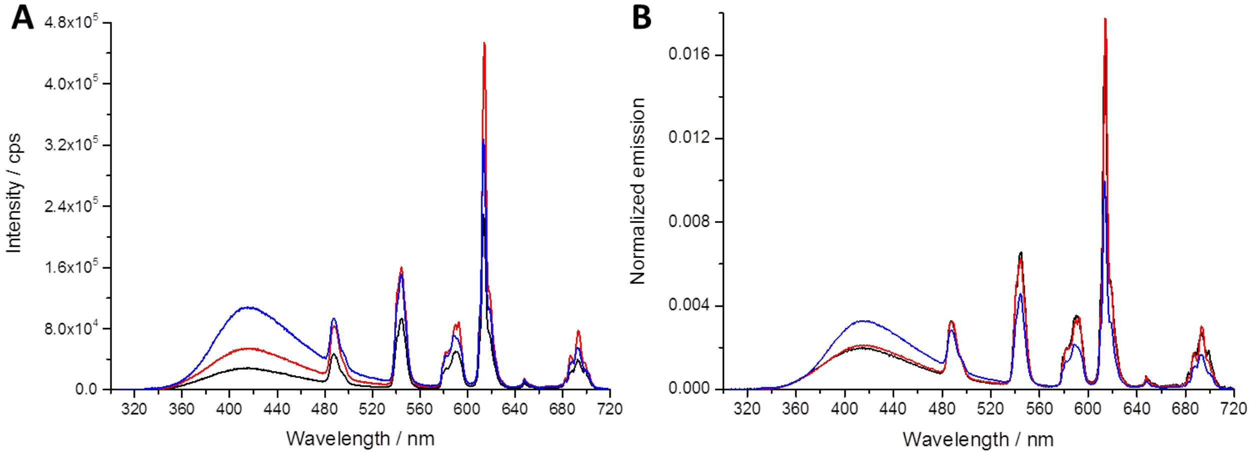

Fig. S25 (A) Fluorescence emission spectra of **tdt**[Eu(EDTA)][Tb(EDTA)]$_2$ in MeOH with [**tdt**] = 12 (—), 25 (—) and 39 μM (—) upon excitation at 270 nm, (B) corresponding fluorescence emission spectra normalised by the area.

Table S5 CIE (x,y) coordinates and blue-to-red intensity ratio (I_{tdt} / I_{Eu}) of the light emitted by **tdt**[Eu(EDTA)][Tb(EDTA)]$_2$ in MeOH as a function of the excitation wavelength (λ_{ex} = 260→340 nm); [**tdt**] = 12, 25 and 39 μM, T = 298 K.

λ_{ex} / nm	260	270	280	290	300	320	340
12 μM CIE (x,y)	(0.303,0.241)	(0.305,0.243)	(0.302,0.232)	(0.195,0.127)	(0.190,0.123)	(0.186,0.120)	(0.190,0.131)
25 μM CIE (x,y)	(0.380,0.313)	(0.383,0.316	(0.342,0.270)	(0.249,0.179)	(0.236,0.165)	(0.232,0.159)	(0.257,0.188)
39 μM CIE (x,y)	(0.386,0.321)	(0.392,0.327)	(0.341,0.272)	(0.230,0.162)	(0.207,0.137)	(0.201,0.128)	(0.216,0.148)
I_{tdt} / I_{Eu} 12 μM	0.34	0.33	0.55	1.52	1.47	1.59	1.19
I_{tdt} / I_{Eu} 25 μM	0.12	0.12	0.17	0.42	0.46	0.51	0.28
I_{tdt} / I_{Eu} 39 μM	0.14	0.13	0.22	0.80	1.12	1.32	0.74

Please do not adjust margins

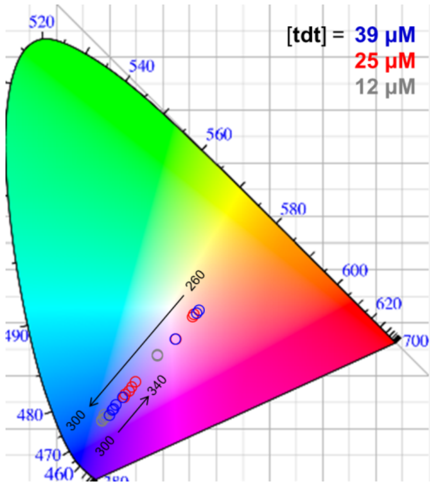

Fig. S26 CIE-1931 chromaticity diagram for **tdt**[Eu(EDTA)][Tb(EDTA)]$_2$ in MeOH as a function of the λ_{ex} and **tdt** concentration.

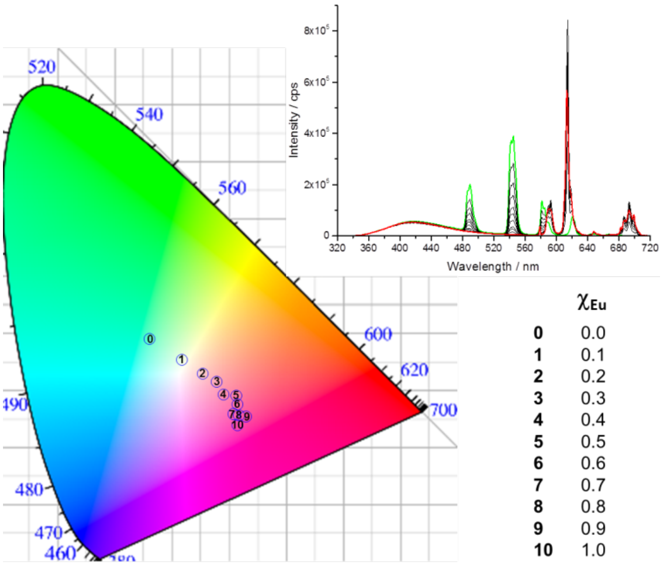

Fig. S27 Fluorescence emission spectra and corresponding CIE-1931 chromaticity diagram for the various **tdt**[Eu(EDTA)]$_x$[Tb(EDTA)]$_{3-x}$ assemblies in methanol as a function of χ_{Eu}, the molar ratio of Eu(EDTA)(H_2O)$_3$; [**tdt**] = 25 μM and λ_{ex} = 270 nm.

Table S6. CIE (x,y) coordinates of the light emitted by **tdt**[Eu(EDTA)]$_{0.3}$[Tb(EDTA)]$_{2.7}$ (χ_{Eu} = 0.1) and **tdt**[Eu(EDTA)]$_{0.45}$[Tb(EDTA)]$_{2.55}$ (χ_{Eu} = 0.15) in MeOH as a function of the excitation wavelength (λ_{ex} = 260→300 nm); [**tdt**] = 25 μM, T = 298 K.

**λ_{ex} / nm**	CIE (x,y) coordinates	
	**χ_{Eu} = 0.1**	**χ_{Eu} = 0.15**
**254**	(0.314,0.316)	(0.316,0.284)
**260**	(0.328,0.340)	(0.335,0.311)
**265**	(0.330,0.346)	(0.343,0.320)
**270**	(0.330,0.344)	(0.340,0.316)
**275**	(0.312,0.317)	(0.321,0.291)
**280**	(0.284,0.271)	(0.283,0.243)
**290**	(0.211,0.166)	(0.193,0.134)
**300**	(0.198,0.146)	(0.176,0.115)

 This journal is © The Royal Society of Chemistry 20xx

Please do not adjust margins

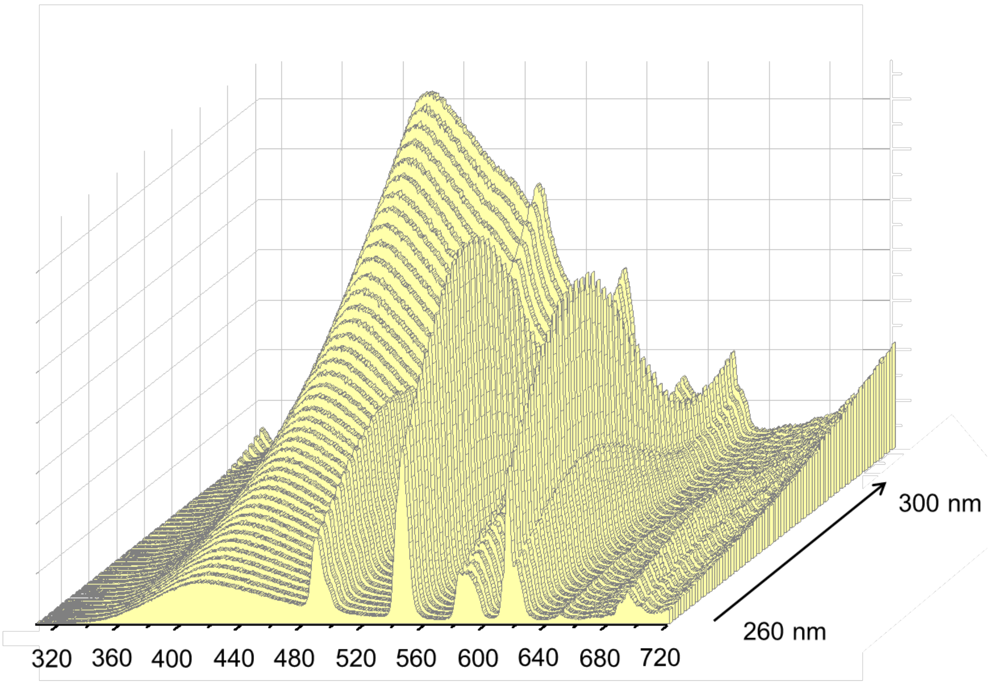

Fig. S28 3D plot displaying the excitation-emission matrix spectra of the luminescent **tdt**[Eu(EDTA)]$_{0.3}$[Tb(EDTA)]$_{2.7}$ assembly; [**tdt**] = 25 μM, λ_{ex} varied from 260 → 300 nm, with spectrum recorded every 1 nm.

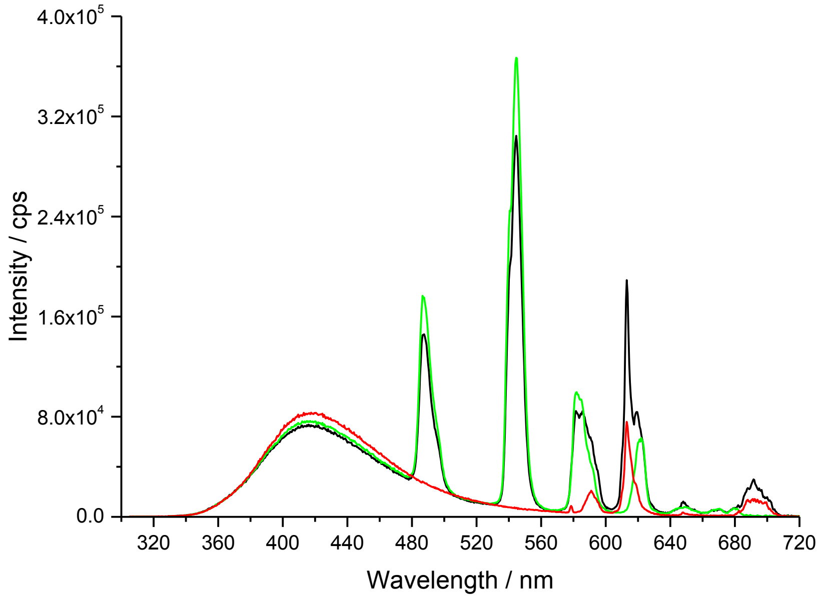

Fig. S29 Comparison of the emission spectrum of three different assemblies of molecular formula **tdt**[Ln^1(EDTA)][Ln^2(EDTA)]$_2$; Ln^1 = Eu, Ln^2 = Gd (—), Ln^1 = Gd, Ln^2 = Tb (—) and Ln^1 = Eu, Ln^2 = Tb (—); [**tdt**] = 25 μM, λ_{ex} = 270 nm.

Please do not adjust margins

Please do not adjust margins

Table S7. Eu(III) and Tb(III) excited state lifetimes for homo- and heterometallic assemblies of general formula **tdt**[Ln^1(EDTA)][Ln^2(EDTA)]$_2$ in MeOH; [**tdt**] = 25 μM, T = 298 K.

Sample	$\lambda_{ex}/\lambda_{an}$	τ_1 / ms	f_1	τ_2 / ms	f_2	$<\tau_{av}>$ / ms
Homometallic						
**tdt**[Eu(EDTA)]$_3$	280/615 nm	0.33(6)	0.26	0.64(4)	0.74	**0.561**
**tdt**[Tb(EDTA)]$_3$	280/545 nm	0.63(2)	0.34	1.28(3)	0.66	**1.053**
Heterometallic						
**tdt**[Eu(EDTA)][Gd(EDTA)]$_2$	280/615 nm	0.40(2)	0.68	0.95(7)	0.32	**0.573**
**tdt**[Eu(EDTA)][Tb(EDTA)]$_2$ [a]	280/615 nm	0.50(1)	0.65	1.23(2)	0.35	**0.754**
**tdt**[Eu(EDTA)][Tb(EDTA)]$_2$	280/545 nm	0.57(6)	0.40	1.13(8)	0.60	**0.906**
**tdt**[Gd(EDTA)][Tb(EDTA)]$_2$	280/545 nm	0.61(3)	0.35	1.24(4)	0.65	**1.017**

[a] as there is a slight overlap between the Eu(III) $^5D_0 \rightarrow {}^7F_2$ and the Tb(III) $^5D_4 \rightarrow {}^7F_3$ transitions, the Eu($^5D_0$) excited state lifetime was also monitored at 696 nm, for which $<\tau_{av}>$ = 0.701 ms

This journal is © The Royal Society of Chemistry 20xx

Please do not adjust margins
